# Multiple Polymorphs and their Relationships in the Potential Ferroelectric Piperazinium Tetrafluoroborate

**DOI:** 10.1021/acs.cgd.5c01210

**Published:** 2025-12-05

**Authors:** Sam Y. Thompson, Ella I. D. Thomson, Faith G. Pritchard, Toby J. Blundell, Jan R. R. Verlet, John S. O. Evans

**Affiliations:** Department of Chemistry, 3057 Durham University, Lower Mount Joy, South Road, Durham DH1 3LE, U.K.

## Abstract

Symmetry-raising phase transitions are essential in the understanding of ferroelectric materials. Here, we investigate the complex polymorphism of piperazinium tetrafluoroborate, identified as a potential ferroelectric by our database search tool, FERROSCOPE. Using powder and single-crystal X-ray diffraction techniques, we have solved the structures of five polymorphs, two of which are newly observed. Space group choices were informed by variable-temperature second-harmonic generation measurements. We extend the structural family to piperazinium perrhenate and solve its room and low temperature structures. Group-subgroup analysis rationalizes the relationship between all phases including those of piperazinium perchlorate previously reported in the literature.

## Graphical abstract

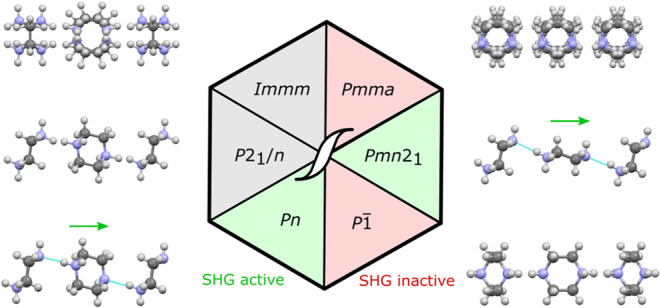

## 1 Introduction

Understanding symmetry-changing phase transitions is essential in the search for new functional materials. Ferroelectric materials, for example, are principally understood with relation to symmetry-breaking, where distortions of a crystal structure reduce the symmetry to a polar space group, enabling spontaneous polarization and switchable ferroelectric domains. Ferroelectrics have uses including in memory devices, sensors and energy harvesting.^1,2^ Molecular ferroelectrics have emerged as potential replacements for conventional inorganic ferroelectrics (BaTiO_3_, Pb­(Zr,Ti)­O_3_) due to their low toxicity and solution processability for thin film applications.^3–5^

The search for new molecular ferroelectrics has historically involved trial-and-error testing of polar structures informed by chemical intuition. This paper describes a structural investigation of a potentially ferroelectric molecular crystal, piperazinium tetrafluoroborate (PipBF_4_, CSD refcode: WAQBOH),^6^ identified as a candidate ferroelectric by our recently published program FERROSCOPE.^7^ FERROSCOPE searches for potential symmetry-raising phase transitions in the Cambridge Structural Database (CSD) as a proxy for ferroelectric switching, as described elsewhere,^8^ and identified PipBF_4_ as an interesting candidate material using the search parameters previously described.^7^

At room temperature, PipBF_4_ crystallizes in polar space group *Pn* with cell parameters *a* = 8.4324(10) Å, *b* = 9.1762(11) Å, *c* = 9.5654(12) Å and β = 98.418(12)° and was solved using single crystal X-ray diffraction (SXRD). This polymorph was labeled as Form IV^6^ and was shown to undergo three reversible phase transitions on warming/cooling at 364/349 K, 386/372 K and 421/411 K by differential scanning calorimetry (DSC), thermogravimetric analysis (TGA), dilatometric measurements and powder X-ray diffraction (PXRD). The observed phases were labeled IV → I with increasing temperature, however the diffraction data were of insufficient quality to solve the higher temperature structures.

In this article, we reanalyze the high temperature polymorphs of PipBF_4_. The various forms observed are summarized in Figure 1b. We solve the structure of polymorphs III and II using SXRD and the structure of I using PXRD with space-group choices informed by second-harmonic generation (SHG) data to confirm noncentrosymmetric cases. Two new polymorphs, V and VI, were observed and their structures have been solved from PXRD data. Using the ISOTROPY language^9^ of group-subgroup relationships and a hypothetical parent supergroup, we describe the relationships between these structures as well as the three known structures of the related piperazinium perchlorate (PipClO_4_).^10^ We extended this structural family by synthesizing the perrhenate analogue (PipReO_4_) which is isostructural to PipClO_4_–III at high temperature, and exhibits a new structure type below 148 K. Both are included in the group-subgroup analysis. Polymorphs are labeled to be consistent with the literature. This means that polymorph labels do not define isostructurality across compositions. The only shared polymorph pair confirmed is PipClO_4_–III = PipReO_4_–I. However, we suspect that PipBF_4_–I and PipClO_4_–I are isostructural.

**Figure 1:**
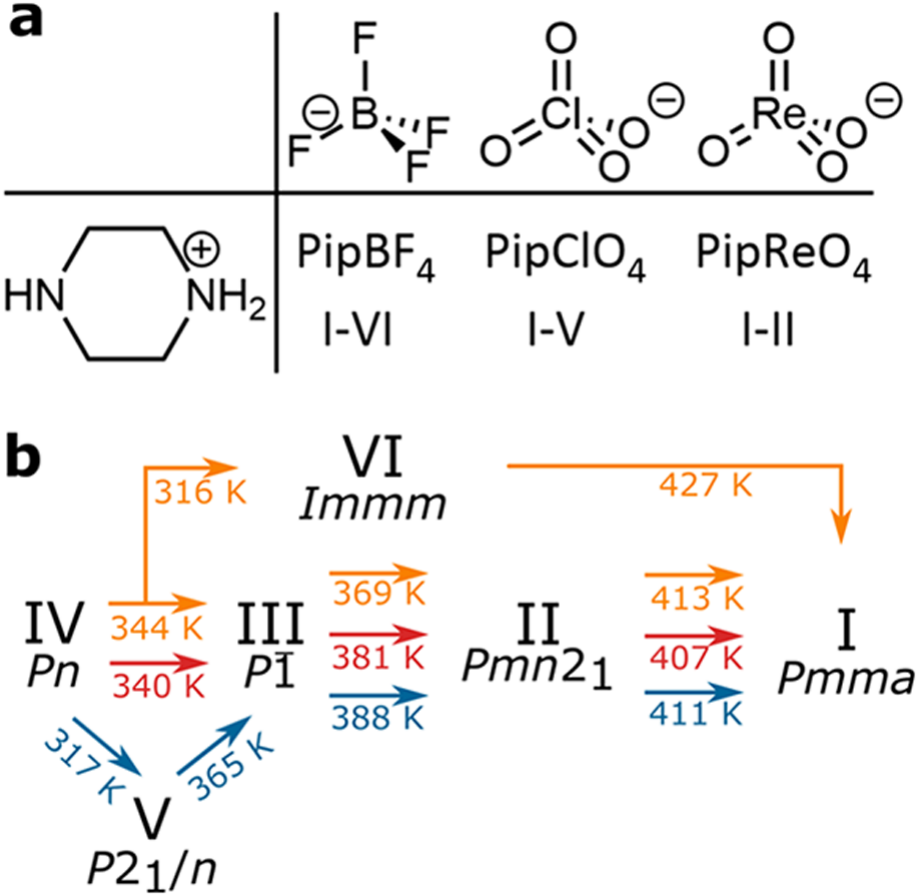
(a) Molecular structures of piperazinium tetrafluoroborate, piperazinium perchlorate and piperazinium perrhenate. (b) Schematic chart showing the transitions observed in Figure 3 (red), Figure 6a (blue) and Figure 6c (orange).

## 2 Experimental Section

### 2.1 Synthesis

A literature method^6^ was adapted to synthesize PipBF_4_. Piperazine (2.50 g, 29 mmol, Fisher Scientific, 99%) was dissolved in deionized water (5 mL) followed by the dropwise addition of a stoichiometric amount of tetrafluoroboric acid (1.8 mL, 29 mmol, Aldrich, 48 ± 2% in H_2_O). After 2 weeks of solvent evaporation, colorless plate-like crystals were produced. Successful synthesis was confirmed through Rietveld refinement using the literature structure (see Figure 5 later).

To synthesize PipReO_4_, piperazine (0.215 g, 2.5 mmol) was dissolved in water (5.0 mL), followed by the dropwise addition of a stoichiometric amount of perrhenic acid (0.29 mL, 2.5 mmol, Fisher Scientific, 76.5 wt % in H_2_O, 99.99% pure). The solution was left to evaporate for 7 days until colorless plate-like single crystals were obtained.

Attempts to extend this structural family to gallium and aluminum analogues failed to produce the target compounds.

Two new crystal structures (PhPipBF_4_ and PhBorox) were discovered during this investigation and are detailed in the SI.

### 2.2 Powder X-ray Diffraction (PXRD)

Variable temperature PXRD data were collected using a Bruker D8 ADVANCE Cu Kα diffractometer, equipped with a LYNXEYE detector and an Oxford Cryosystems Cryostream Plus device. The sample was loaded into a 0.7 mm external diameter borosilicate capillary to a length of 30 mm and sealed. The sample was spun at 10 rotations a minute during the measurements. Data were analyzed using TOPAS-Academic.^11–13^ Cell parameters, background polynomial, sample height correction, axial peak asymmetry, a TCHZ peak shape with minor *hkl-*anisotropy and a single isotropic temperature factor were allowed to refine. Cryostream temperatures were calibrated using a 1:1 ratio of Al and Si powders, which have significantly different thermal expansion coefficients. Diffraction patterns were collected between 80 and 500 K, and the true sample temperatures were determined by comparing experimental cell parameters to known thermal expansion data.^14–16^

### 2.3 Single Crystal X-ray Diffraction (SXRD)

SXRD data were collected with a Bruker D8 VENTURE diffractometer (PHOTON III C7MM CPAD detector, ImS-microsource, focusing mirrors) mainly using Mo Kα radiation. Cu Kα was used in the PipBF_4_–III experiment. Sample temperatures were set using an Oxford Cryosystems Cryostream 700+ device. Crystal structures were solved and refined within the Olex2 software package.^17^ Carbon H atoms were placed in calculated positions and refined in riding mode. Nitrogen H atoms were located from difference maps, where possible.

### 2.4 Second-Harmonic Generation (SHG)

An in-house SHG experimental setup was adapted to study samples at temperatures up to 500 K. The experiment is loosely based on the Kurtz and Perry method^18^ widely applied in materials science.^19^ A crystalline sample of PipBF_4_ was very lightly ground and packed into a 0.7 mm borosilicate capillary which was mounted on a goniometer head. Incident light was produced with a pulsed laser (CARBIDE, Light Conversion) with infrared wavelength 1028 nm and focused on the sample capillary. Green (514 nm) emitted SHG light was reflected onto a photomultiplier tube (PMT) detector (Figure S4). An Oxford Cryosystems Cryostream Plus system was aligned over the capillary such that the distance between the nozzle and the incident laser beam is the same as in PXRD measurements. This ensures that the PXRD temperature calibration is also valid for this experiment. Variable temperature SHG data were collected between 292 and 430 K with a ramp rate of 6 K min^–1^.

## 3 Results and Discussion

### 3.1 Crystal Structure and Symmetry-Raising Predictions of PipBF_4_–IV

The room temperature structure of PipBF_4_ is deposited in the CSD with refcode WAQBOH. It crystallizes in space group *Pn* with cell parameters *a* = 8.4324(10) Å, *b* = 9.1762(11) Å, *c* = 9.5654(12) Å and β = 98.418(12)° at 295 K.^6^ We repeated the SXRD analysis and found this *Pn* phase persists down to at least 120 K. We found a similar structural model with cell parameters *a* = 8.3950(5) Å, *b* = 9.1069(6) Å, *c* = 9.4858(6) Å and β = 98.852(2)° at 120 K. The structure contains chains of piperazinium running parallel to the *c*-axis, linked by hydrogen bonds between the amine groups of adjacent cations as seen in Figure 2. Hydrogen atoms within the chains order such that a net polarization is present. BF_4_^–^ tetrahedral anions fill the gaps between the cation chains with one of their 3-fold axes roughly parallel to the *c*-axis. We model these as rotationally disordered across two equally occupied orientations in contrast to the single orientation model in the CSD entry. All the structures discussed in this article maintain this same basic arrangement of cations and anions. In PipBF_4_–IV, the piperazinium planes along the chains alternate in an upright-face-on-upright pattern when viewed along the *a*-direction. This configuration is labeled Chain 2. A second configuration, which we label Chain 1, has the pattern upright-flat-upright and is found in other structures of this family (Figure 2b).

**Figure 2:**
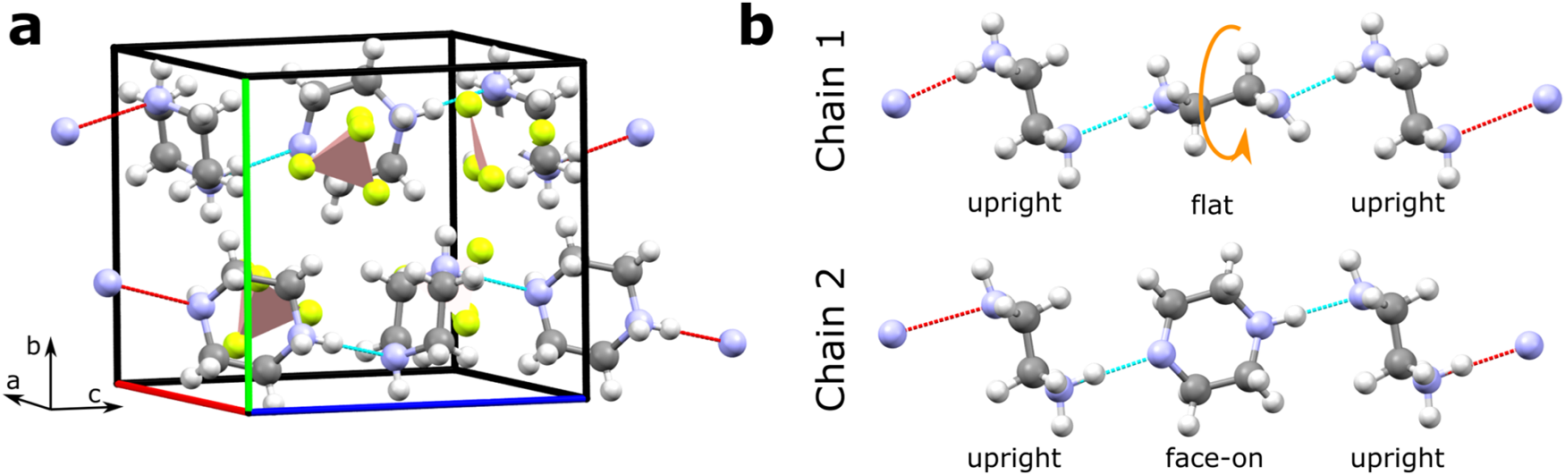
(a) Unit cell of PipBF_4_–IV at room temperature which features antiparallel piperazinium chains of the Chain 2 configuration. (b) The two observed chain types in the salts discussed. The orange arrow represents a 90° rotation about the chain direction that interconverts flat and face-on piperaziniums. Colored spheres in this publication represent nitrogen (light blue), carbon (gray), boron (pink), hydrogen (white), fluorine (yellow), chlorine (green) and rhenium (dark blue) atoms.

Our SXRD solution was used as a model in a Rietveld refinement against high-quality PXRD (43 summed 25 min scans recorded at room temperature) to confirm the purity of the bulk sample. An *R*_wp_ of 5.63% and a good fit were achieved as shown later in Figure 5. The unfitted peak at 14.5° does not discontinuously change through subsequent phase transitions so we believe it is due to a small impurity.

FERROSCOPE predicts that PipBF_4_ may undergo a symmetry-raising transition to *P*2_1_/*n*. This symmetry is achieved by the hydrogen atoms becoming disordered within the chain hydrogen bonds. WAQBOH was also identified as a potential ferroelectric material by Dypvik Sødahl et al. using their globularity approach.^20^ They predict a significant spontaneous polarization of 8.1 μC/cm^2^.

### 3.2 Observations of Structural Phase Transitions

To corroborate the phase transition observations of Wojtas et al., variable temperature PXRD data were collected on PipBF_4_ between 298 and 430 K on warming and cooling (Figure 3). On warming, three phase transitions were evidenced by abrupt changes in the Bragg peaks confirming their discontinuous nature. All transitions are reversible upon cooling, albeit with significant transition temperature hysteresis. Wojtas et al. observed phase transition temperatures of (warming/cooling) 364/349 K, 386/372 K and 421/411 K; in our experiments these were observed at 340/312 K, 381/346 K and 407/384 K. The phase transitions in our experiment were all observed at lower temperatures. This is due to the heating rate differences between the DSC (10 K min^–1^) and PXRD measurements (0.16 K min^–1^). A second PXRD run was performed over a narrow 2θ range (22–24°) so the temperature ramp rate could be increased to 6 K min^–1^ to match our SHG experiment described later. The transition temperatures here were observed as 351/306 K, 380/347 K, 411/383 K.

**Figure 3:**
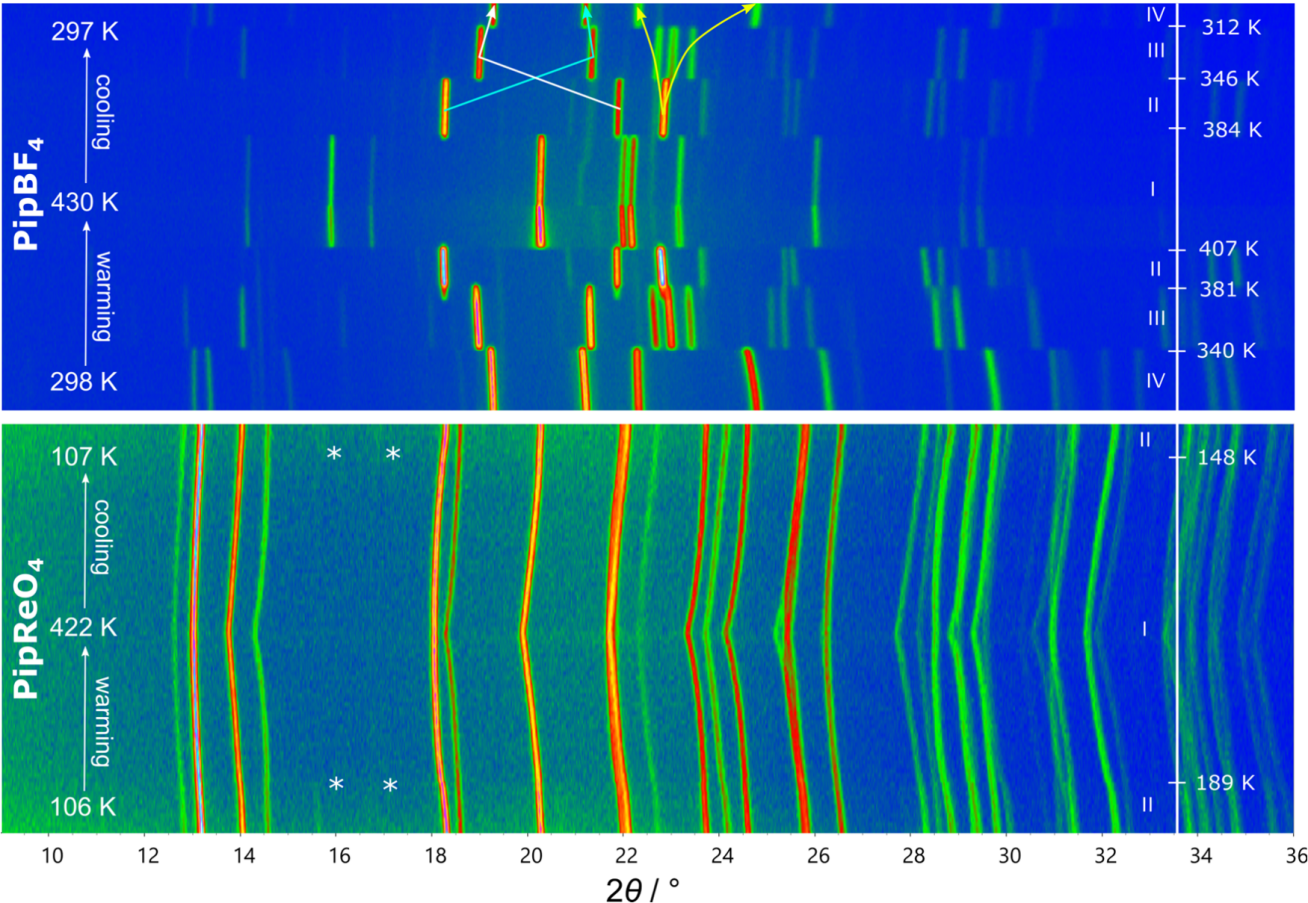
Surface plot of variable temperature PXRD data on warming and cooling between 298 and 430 K for PipBF_4_ (upper) and between 106 and 422 K for PipReO_4_ (lower). Diffraction intensities are shown by an artificial color map from low intensity blue to high intensity orange. The labeled peaks and colored arrows are discussed in the text.

VT-PXRD data were also collected on PipReO_4_ between 106 and 422 K to look for possible phase transitions. Upon cooling from 400 K, no transitions are observed until two weak additional peaks appear at 15 and 17° (marked with * in Figure 3) at around 148 K. On warming, the transition is reversible. As there is significant hysteresis in the phase transition temperatures (189/148 K) and the weak additional peaks appear with only minor changes in the original peaks, this is likely a discontinuous phase transition to a structure with a supercell of the room temperature unit cell; this is discussed further below.

For both PipBF_4_ and PipReO_4_, Rietveld refinements were performed at every temperature to extract unit-cell parameters using the structural models described in Sections 3.1 and 3.3–3.5. The thermal expansion represented as *V*/*Z* on warming are plotted in Figures 4a and S1, respectively. For PipBF_4_, jumps in *V*/*Z* are observed at each phase transition temperature and correspond to the discontinuous diffraction pattern changes in Figure 3. As expected from the subtle diffraction pattern change, no discontinuity is observed in *V*/*Z* at the phase transition temperature (189 K) of PipReO_4_, although a slight gradient change does occur (Figure S1).

**Figure 4:**
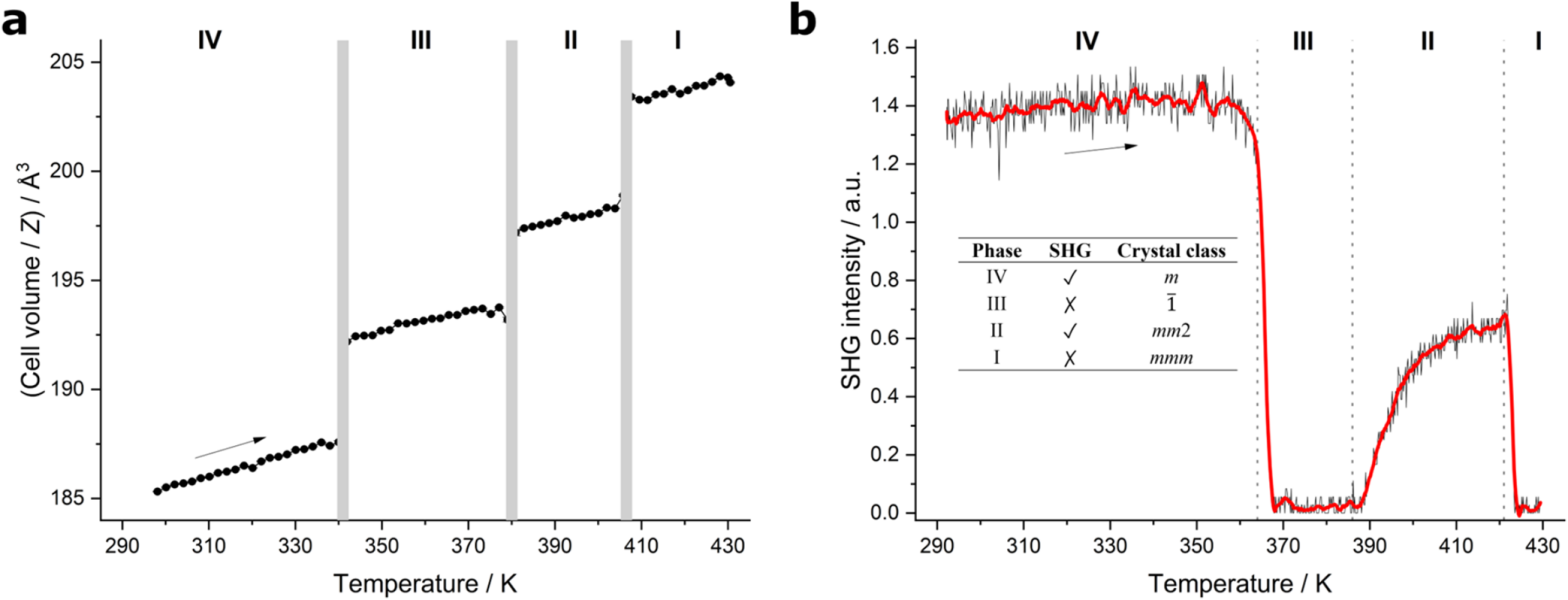
(a) Thermal expansion of the *Z*-normalized volume of PipBF_4_ on warming. (b) SHG intensity of PipBF_4_ with increasing temperature. Vertical dotted lines are DSC phase transition temperatures from the literature.^6^

Variable temperature second-harmonic generation data were collected on PipBF_4_ between 298 and 430 K on warming (Figure 4b). At room temperature, PipBF_4_ exhibits an SHG signal (roughly 0.2 × KDP), in agreement with the noncentrosymmetric space group assignment of *Pn* from SXRD analysis. Upon warming, three phase transitions are evidenced at temperatures consistent with the changes in the diffraction data. A first transition at ∼360 K shows complete loss of SHG intensity suggesting the appearance of a centrosymmetric phase. Between 385 and 410 K, the SHG intensity gradually reappears showing the slow growth of a noncentrosymmetric phase. A second experiment was performed that showed that this process is slow with respect to time rather than temperature: slower-warming through this temperature produced a transition as sharp as the other two transitions. Finally, above ∼420 K, the SHG intensity is lost suggesting the highest temperature phase I is centrosymmetric. This information is used to inform space-group choices in the following sections.

### 3.3 Crystal Structure of PipBF_4_–III

Between 340 and 381 K (on warming), PipBF_4_ adopts a triclinic structure which was solved using SXRD data. A cell with space group *P*1̅ (centrosymmetric in accordance with SHG measurements) was found with cell parameters *a* = 8.0065(5) Å, *b* = 8.0351(6) Å, *c* = 19.1239(12) Å, α = 81.381(5)°, β = 79.057(5)°, γ = 76.226(6)° at 370 K. The cell volume is 1.5 times that of the room temperature PipBF_4_–IV cell. The general arrangement of piperazinium chains and BF_4_^–^ anions is the same as phase IV, however 1/3 of the chains now adopt what we call the Chain 1 configuration with the rest remaining as Chain 2 (Figure 2b) This 2:1 ratio requires the 1.5 × cell volume change. The three BF_4_^–^ anions in the asymmetric unit all show some degree of disorder across two rotational orientations. This is analogous to results from molecular dynamics on NaBF_4_. These demonstrate that at high temperature BF_4_^–^ tetrahedra rapidly rotate between orientations observed in the average structure.^21^

This model was used in a Rietveld refinement against high-quality PXRD data (157 summed 25 min scans recorded at 357 K) achieving an *R*_wp_ of 3.29% (Figure 5) and a good fit. Small, unfitted peaks at 19.5 and 21.9° are a result of an unidentified impurity. These peaks are absent in other data collections on III. The large structural difference between III and IV explains the distinct difference in the diffraction patterns, and the higher number of peaks observed for phase III over phase IV results from the large triclinic (lower symmetry) cell compared to the smaller monoclinic cell. We note that this is an example of symmetry-lowering on warming This is relatively rare but has been observed previously in molecular ferroelectrics.^22,23^ In fact, the first molecular ferroelectric, Rochelle salt, exhibits a symmetry-lowering phase transition from *P*2_1_2_1_2 to *P*2_1_ at 255 K on heating.^24,25^

**Figure 5:**
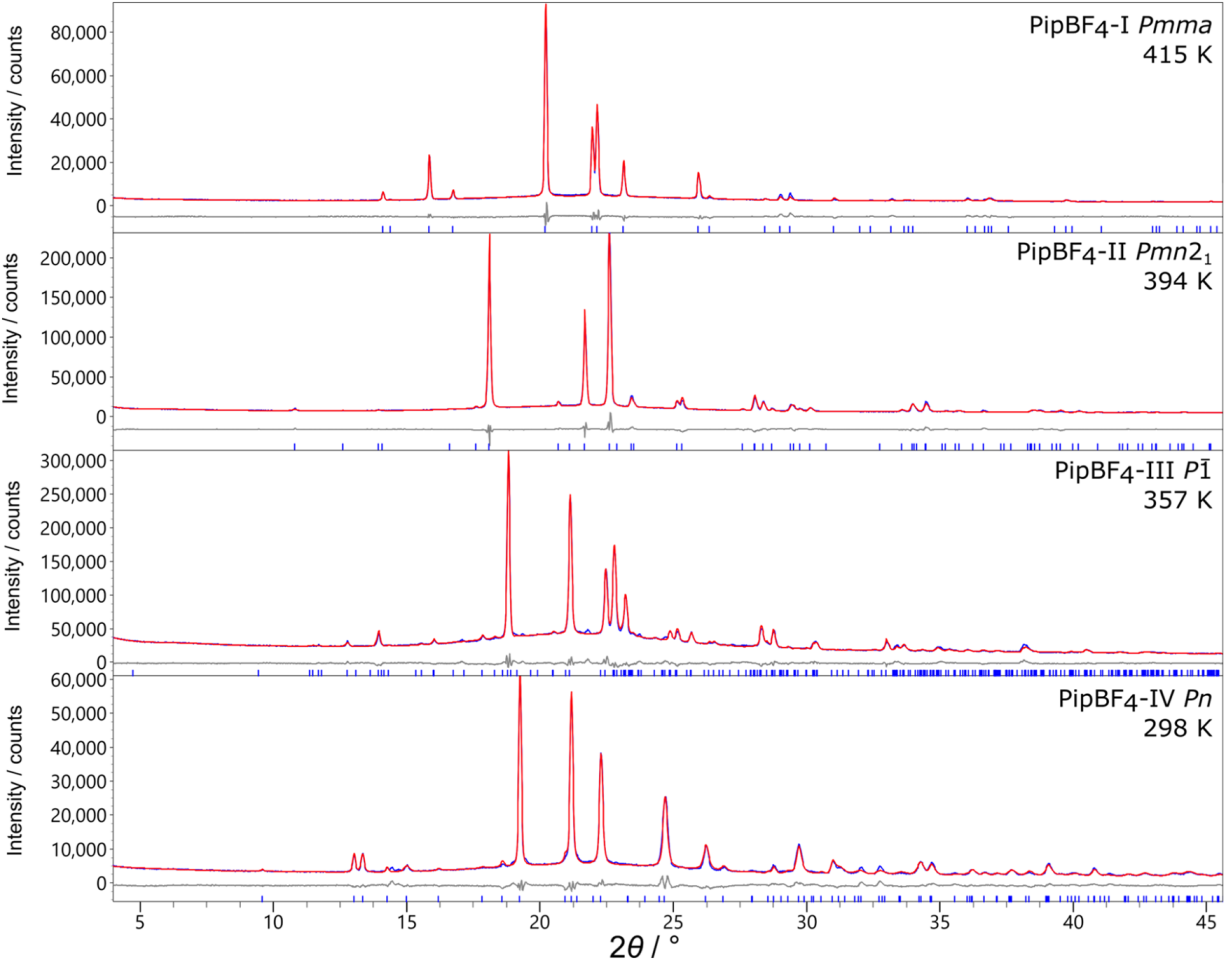
Powder X-ray diffraction data (blue) with the calculated patterns from Rietveld refinements (red) and difference curves (gray). Phases IV–II use the SXRD models; I was solved from the powder data.

### 3.4 Crystal Structure of PipBF_4_–II

Between 381 and 407 K (on warming), PipBF_4_ adopts an orthorhombic structure with space group *Pmn*2_1_ and cell parameters *a* = 9.7292(8) Å, *b* = 8.1355(7) Å, *c* = 9.9939(8) Å as solved by SXRD at 385 K. All chains now adopt the Chain 1 configuration. Due to the high temperature of the data collection, the diffraction data is not of sufficient quality to be certain of hydrogen positions or the extent of the BF_4_^–^ disorder. However, we are confident in the polar nature of this phase as, perpendicular to the *c*-axis, there are no glide planes (reflections that would be systematically absent in the presence of glides are clearly observed) or mirror planes (these would require significant disorder in the piperaziniums which is not observed). SHG measurements confirm this phase is noncentrosymmetric. This suggests the hydrogen atoms remain localized on a single nitrogen atom and could potentially still participate in a ferroelectric switching mechanism. The BF_4_^–^ anions still have a 3-fold axis of molecular symmetry parallel to the *c*-axis however the data suggest that rotational disorder about this axis occurs at this temperature. Our model also gives a good fit against high-quality PXRD data (19.5 h scan) as seen in Figure 5 achieving an *R*_wp_ of 4.46%.

### 3.5 Crystal Structure of PipBF_4_–I

Above the final phase transition temperature, the crystal shattered and a structure for phase I could not be determined by SXRD. Structure solution was performed directly from powder diffraction data. A high-quality data set was recorded over 6 h at 446 K. Indexing produced an orthorhombic unit cell with dimensions *a* = 5.2926 Å, *b* = 6.2691 Å, *c* = 12.3020 Å with systematic absences consistent with *Pmc*2_1_ or *Pmma*. The SHG measurements indicate that the space group of PipBF_4_–I is likely centrosymmetric so *Pmma* was chosen. A rigid body of piperazinium was positioned at (0, 0.5, 0.5). and BF_4_^–^ anions were positioned at (0.25, 0, *z*), where *z* is a refinable parameter. Both ions were given three degrees of rotational freedom. Rigid body rotations and translations were randomized and refined for 100,000 least-squares iterations. The best structure was used in a Rietveld refinement giving an *R*_wp_ of 5.2% and the fit is included in Figure 5. The resultant structure contains BF_4_^–^ anions disordered across 4 orientations which appears representative of isotropic free rotation. The piperazinium cations are disordered across 4 sites such they appear to represent the molecule rotating in its pseudoplane, and the N–H hydrogens must be disordered by symmetry. It is not possible to determine whether there is dynamic free rotation or a restricted hopping from powder data alone.

### 3.6 Crystal Structure of PipBF_4_–V

As well as solving all the structures observed by Wojtas, other polymorphs of PipBF_4_ were identified during some variable temperature powder data collections. These appeared only over narrow temperature ranges and the behavior was not reproducible. For example, room temperature phase IV typically transitions to phase III at 340 K, however in the experiment shown in Figure 6, a transition occurred to a previously unobserved phase at 317 K. This phase (which we label V) is closely related to phase IV, as seen by the continuity of the strongest peaks through the phase transition (19.2, 21.0, 28.6 and 30.9°). The (112̅) (22.7°) and (112) (23.4°) peaks of phase IV approach one another but do not merge, suggesting phase V has a monoclinic cell with a β angle closer to 90° than phase IV. This phase was expected to have the *P*2_1_/*n* structure that was predicted by FERROSCOPE to be the paraelectric phase of structure IV. The FERROSCOPE *P*2_1_/*n* structure was therefore used as a starting model for Rietveld refinement. To achieve this symmetry, the N–H···N hydrogen bonds must become centered with the hydrogen atoms shared equally between both nitrogen atoms. The BF_4_^–^ rigid body was given three degrees each of rotational and translation freedom. These parameters were refined as in Section 3.5. This gave a reasonable fit to the data as seen in Figure 6b, though a couple of weak peaks are unexplained. Given the data quality, we cannot be sure if these are from V or a small quantity of another polymorph. The final unit-cell parameters were: *a* = 8.449(2) Å, *b* = 9.235(3) Å, c = 9.7842(19) Å and β = 92.276(14)°. While this is a reasonable structural model, we do not consider it definitive. In this experiment phase V transitioned to phase III at 365 K and then followed the expected phase sequence upon subsequent warming as in Figure 3.

**Figure 6:**
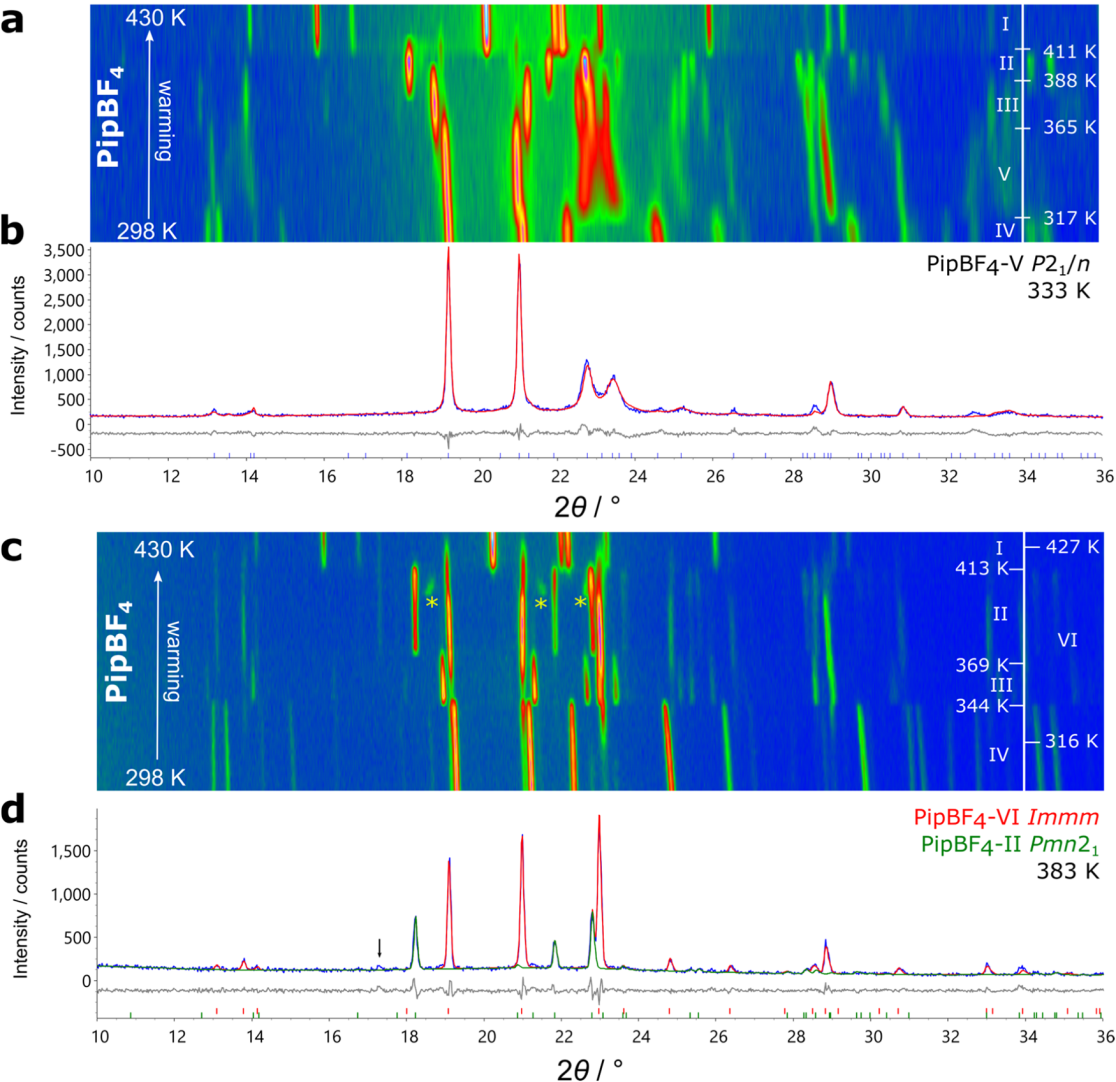
Surface plots of variable temperature PXRD data on warming PipBF_4_ between 298 and 430 K with the observations of polymorphs (a) V and (c) VI. The peaks labeled with yellow asterisks indicate a transient unsolved phase. (b) A single pattern (blue) taken from the variable temperature experiment of (a), showing the calculated pattern from the Rietveld refinements (red) and the difference curve (gray) at 333 K. (d) A single pattern (blue) taken from the variable temperature experiment of (c), showing the calculated pattern from the multiphase Rietveld refinements with phases VI (red) and II (green) and the difference curve (gray) at 383 K. The black arrow labels an impurity peak.

### 3.7 Crystal Structure of PipBF_4_–VI

The separate heating experiment shown in Figure 6c reproduced the phase transition sequence observed in Figure 3 with reasonable agreement between the transition temperatures. However, a minor secondary phase present at room temperature appears to seed a new phase of PipBF_4_ which was present between 316 and 427 K. We label this new phase VI and, like V, it is closely related to IV with strong peaks at 19.1 and 21.0° appearing in the patterns of all three phases. The single peak at 23.0° is likely due to exact overlap of the (112̅) and (112) peaks observed in phases IV and V and indicates that phase VI is orthorhombic (Table 1).

**Table 1:** Unit-Cell Parameters for the Discussed Structures^a^

phase	space group	temp/K	solution cell parameters/Å	transformation matrix to PipBF_4_–IV cell	PipBF_4_–IV cell parameters/Å	volume per molecule/Å^3^	*Z*, *Z*’
PipBF_4_–IV	*Pn*	120	*a* = 8.3950(5)		*a* = 8.3950(5)	179.14	4, 2
			*b* = 9.1069(6)		*b* = 9.1069(6)		
			*c* = 9.4858(6)		*c* = 9.4858(6)		
			β = 98.852(2)°		β = 98.852(2)°		
PipBF_4_–V	*P*2_1_/*n*	333	*a* = 8.449(2)	$\left(\begin{array}{lll} 1 & 0 & 0\\ 0 & 1 & 0\\ 0 & 0 & 1 \end{array}\right)$	*a* = 8.449(2)	190.70	4, 1
			*b* = 9.235(3)		*b* = 9.235(3)		
			*c* = 9.7842(19)		*c* = 9.7842(19)		
			β = 92.276(14)°		β = 92.276(14)°		
PipBF_4_–III	*P*1̅	370	*a* = 8.0065(5)	$\left(\begin{array}{lll} -1 & -2 & 1\\ -1 & -2 & -1\\ -1 & 1 & 0 \end{array}\right)$	*a* = 3 × 8.3614(4)	194.35	6, 3
			*b* = 8.0351(6)		*b* = 3 × 9.3959(6)		
			*c* = 19.1239(12)		*c* = 9.9011(9)		
			α = 81.381(5)°		α = 88.572(8)°		
			β = 79.057(5)°		β = 91.287(6)°		
			γ = 76.226(6)°		γ = 89.597(6)°		
PipBF_4_–II	*Pmn*2_1_	385	*a* = 9.7292(8)	$\left(\begin{array}{lll} 1 & 0 & 0\\ 0 & 1 & 0\\ 0 & 0 & 1 \end{array}\right)$	*a* = 9.7292(8)	197.76	4, 1
			*b* = 8.1355(7)		*b* = 8.1355(7)		
			*c* = 9.9939(8)		*c* = 9.9939(8)		
PipBF_4_–I	*Pmma*	415	*a* = 12.3084(9)	$\left(\begin{array}{lll} 1 & 1 & 0\\ 0 & 0 & 1\\ 2 & -2 & 0 \end{array}\right)$	*a* = 2 × 8.7881(2)	204.47	2, 1
			*b* = 5.2959(4)		*b* = 0.5 × 10.5918(2)		
			*c* = 6.2734(6)		*c* = 8.7881(2)		
					β = 91.09935°		
PipBF_4_–VI	*Immm*	383	*a* = 8.4621(16)	$\left(\begin{array}{lll} 1 & 0 & 0\\ 0 & 1 & 0\\ 0 & 0 & 1 \end{array}\right)$	*a* = 8.4621(16)	193.4	4, 2
			*b* = 9.2890(19)		*b* = 9.2890(19)		
			*c* = 9.8423(15)		*c* = 9.8423(15)		
PipReO_4_–I	*P*1̅	298	*a* = 7.6478(8)	$\left(\begin{array}{lll} -1 & -1 & 1\\ -1 & -1 & -1\\ 1 & -1 & 0 \end{array}\right)$	*a* = 2 × 8.9287(6)	210.53	4, 2
			*b* = 8.4670(8)		*b* = 2 × 9.8313(6)		
			*c* = 13.6549(13)		*c* = 9.6986(10)		
			α = 88.233(4)°		α = 88.979(7)°		
			β = 82.646(4)°		β = 97.633(8)°		
			γ = 73.807(4)°		γ = 86.708(8)°		
PipReO_4_–II	*P*1̅	120	*a* = 12.7437(8)	$\left(\begin{array}{lll} -1 & 5 & 1\\ 1 & 1 & 2\\ 3 & 3 & 0 \end{array}\right)$	*a* = 6 × 8.8520(5)	204.83	12, 6
			*b* = 15.1771(9)		*b* = 6 × 9.7123(8)		
			*c* = 15.6866(9)		*c* = 3 × 9.6280(7)		
			α = 103.781(2)°		α = 88.918(5)°		
			β = 110.959(2)°		β = 97.270(3)°		
			γ = 108.814(2)°		γ = 86.571(6)°		

a Transformation matrices are provided between the unit cells of the newly reported structures and the previously reported phase IV, and cell parameters following transformation are given.

To solve the structure of VI, a 383 K pattern was used in which both VI and II were present. First, a two-phase refinement was performed with phase II described by a structural model (with a [112] March-Dollase^26^ preferred orientation correction) and phase VI with a Pawley^27^ model based on the unit cells of phase IV/V with β = 90°. Space groups *Pnnm*, *Pnnn* and *Immm* would be compatible with different disorder models of the piperazinium chains in phase IV. Pawley fits in all three space groups were similar, and *Immm* was selected as the highest symmetry possibility that explained all the experimentally observed reflections. An *R*_wp_ of 10.6% was achieved. The small unfitted peak at 17.3° (black arrow in Figure 6d) is a minor impurity, as evidenced by its retention in the data at subsequent phase transitions.

A disordered structural model was created for Phase VI based on the structure of phase IV. In this *Immm* model, piperazinium chains lose their directionality such that the parallel or antiparallel chains observed in other phases become equivalent in VI. As the cell parameters are similar to that of phase IV, all chains are expected to remain in the Chain 2 configuration. The upright piperaziniums are then disordered across two sites and the face-on piperaziniums across four sites. A rigid body of BF_4_^–^ was added to the structure and given three degrees of rotational freedom and one degree of translational freedom along the [100] direction. Site occupancies were set to reflect the molecular disorder and that the hydrogen atoms within the hydrogen bonds are disordered. The BF_4_^–^ rotations and translation were allowed to refine as in phase V. Preferred orientation of phase VI was modeled using a [200] March-Dollase correction leading to the Rietveld fit shown in Figure 6d. The nearest B–B intermolecular distance between BF_4_^–^ groups refined to 4.11 Å, similar to that observed in phase IV (4.03 Å). The symmetry of the space group results in 4 orientations of the BF_4_^–^ anion which appear to represent rotational disorder about the [001] direction. The final cell parameters refined to *a* = 8.4621(16) Å, *b* = 9.2890(19) Å and *c* = 9.8423(15) Å.

The peaks that appear at 397 K labeled by the yellow asterisks at 18.5, 21.5 and 22.6° in Figure 6c are indicative of another new phase (we rule out sample decomposition as on continued heating they disappear and pure phase I is formed). The data quality is not sufficient to solve this structure, however the positions of the peaks between the (200) and (020) reflections of phases II and VI suggest that it might be another polymorph of PipBF_4_ with *a* and *b* cell parameters with intermediate values to those of phases II and VI. This may suggest the presence of a mixture of Chains 1 and 2 as in phase III.

### 3.8 Crystal Structure of PipReO_4_–I

We found no evidence in the literature for previous structural characterization of PipReO_4_, so SXRD was used for structure solution. At room temperature, a triclinic structure in space group *P*1̅ was found with cell parameters *a* = 7.6478(8) Å, *b* = 8.4670(8) Å, *c* = 13.6549(13) Å, α = 88.233(4)°, β = 82.646(4)°, γ = 73.807(4)°. The structure is isostructural with that of PipClO_4_–III.^10^ Experimental and calculated powder patterns of the bulk sample from which the crystal was chosen are shown in Figure S2.

### 3.9 Crystal Structure of PipReO_4_–II

Upon cooling below 148 K, PipReO_4_–I undergoes a phase transition to a structure with a significantly larger triclinic cell. SXRD analysis at 120 K gave a *P*1̅ structure with cell parameters *a* =12.7437(8) Å, *b* = 15.1771(9) Å, *c* = 15.6866(9) Å, α = 103.781(2)°, β = 110.959(2)°, γ = 108.814(2)°. As discussed later, this cell is 4 × the volume of the room temperature cell. As expected from the minor changes in the powder diffraction patterns either side of the phase transition (Figure 3), only small changes occur in the positions of the piperazinium and rhenate ions. A Rietveld fit using this model at 106 K is shown in Figure S2.

### 3.10 Structural Relationships between All Observed Phases

The structures of all PipBF_4_, PipClO_4_ and PipReO_4_ phases contain hydrogen-bonded chains of piperazinium ions. However, given the wide range of unit cells and space groups observed (Table 1 and Figure 7), it is not straightforward to understand how to relate the various structures. We can, however, do this using the language of isotropy subgroups.^9^ To explain the majority of the structures (all except PipBF_4_–I), we can use the orthorhombic *Immm* structure of phase VI as a parent. This structure features high disorder of both the piperazinium rings and BF_4_^–^ anions. However, to include PipBF_4_–I in the analysis, it is convenient to use a higher-symmetry hypothetical parent with tetragonal space group *P*4/*mmm*. This has still-higher disorder of the piperaziniums and can be thought of as a tetragonally distorted decorated CsI structure (the TiCu-δ structure) with piperazinium and BF_4_^–^ ions on Cs^+^ and I^–^ sites. To help the discussion, equivalent sketches of a single unit cell of each structure are given in Figure 7 and the group-subgroup tree of Figure 8b summarizes the potential transformation pathways between them. When comparing structures across different unit-cell settings, directions based on the unit cells of PipBF_4_–IV will be used.

**Figure 7:**
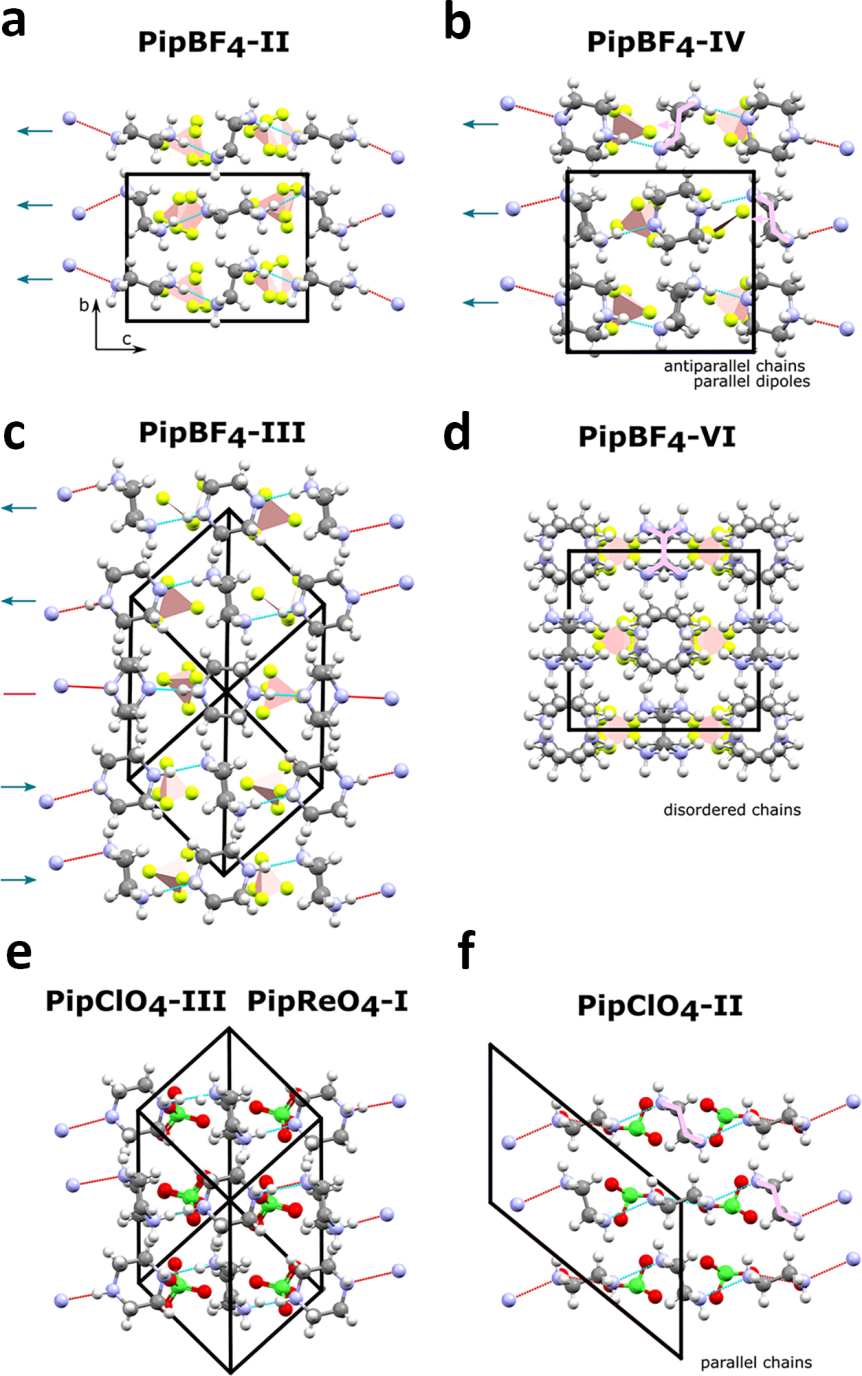
Structural images viewed perpendicular to the piperazinium chains. The distinction between parallel, antiparallel and disordered chains is demonstrated by the pink symbols with the arrows showing the dipole of the corresponding piperazinium. Arrows next to the chains represent the polarization direction of the adjacent chain.

**Figure 8:**
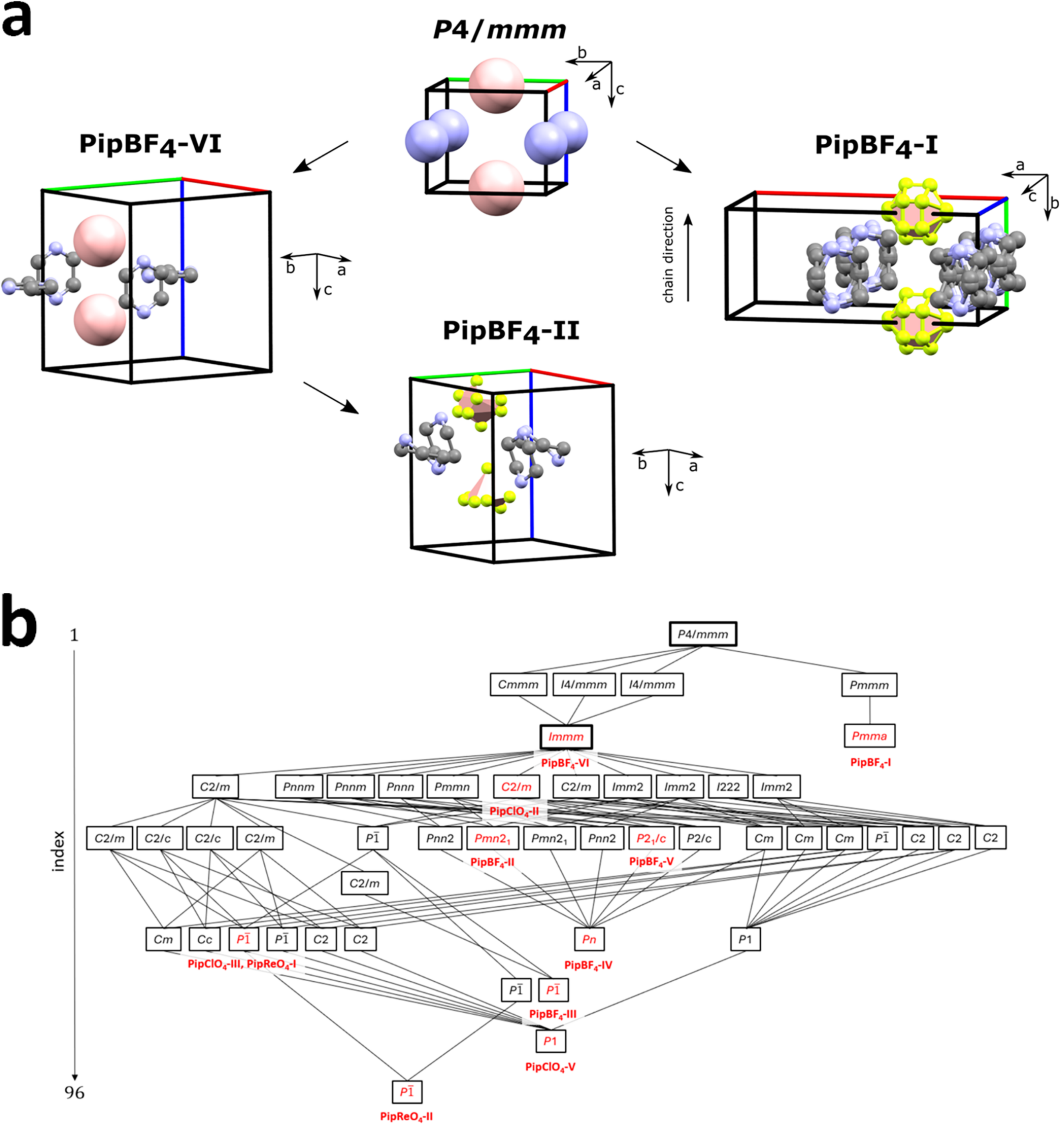
(a) Unit cells of the two structures used to describe the relationship between phases of PipBF_4_. Large atoms indicate isotropic rotation (or a large extent of disorder). (b) Group-subgroup tree containing all known structures of PipBF_4_, PipClO_4_ and PipReO_4_. Red text indicates phases observed experimentally. Bold boxes indicate the parent structures used for relating the different structures.

The PipBF_4_–I structure is derived from the *P*4/*mmm* parent by partial ordering of the piperaziniums driven by strain and ordering modes belonging to the irreducible representation (irrep) Γ_2_^+^ and displacive and ordering modes of the irrep X_2_^+^. The PXRD data suggest that the BF_4_^–^ anions remain spherically disordered. The resulting *Pmma* structure is significantly different to the other structures on the group-subgroup tree. The centroids of the molecular ions are similar to the other phases, however the orientation of piperazinium is distinct. NMR experiments by Wojtas suggested that the hydrogen-bonded chains are broken in phase I.^6^ Due to the large amount of disorder present, we initially agreed with this assessment. However, the piperazinium position refines such that the nitrogen atoms are still able to form N–H···N interactions along the *c*-axis (*b*-axis of the *Pmma* cell). Any hydrogen-bonds must be centered due to the mirror symmetry present. It therefore seems likely the hydrogen is disordered between nitrogen atoms on adjacent piperaziniums. In phase I, the N–N distances between symmetry equivalent nitrogens range from 2.56–2.83 Å compared to 2.87–2.94 Å in phase II. We speculate that what really occurs is that this dynamic hydrogen bond couples with an in-plane rotational disorder of piperazinium which could explain the rise in proton conductivity observed in this phase at higher temperatures.^6^ We also expect that PipClO_4_–I, the structure of which is unknown, will be isostructural with PipBF_4_–I.^10^ Both show a similar entropy of transition (14.5 and 11.2 J mol^–1^ K^–1^, respectively) on their II→I transition, with the small difference likely a result of the preceding child structures being different. PipClO_4_–I also shows proton conductivity at high temperature. Note that the entropy of the transition between PipBF_4_–II and I corresponds to 3.84 microstates which is close to the number of disordered piperazinium sites in the *Pmma* model (4) further supporting this space group choice over *Pmc*2_1_ (2).

The hydrogen-bonded piperazinium chains in all other phases adopt one of two chain configurations. The Chain 1 configuration features an upright-flat-upright sequence of piperaziniums when viewed from the side (Figure 2b). Chain 2 is related to Chain 1 by a ∼90° rotation (orange arrow in Figure 2b) of the flat piperaziniums to a face-on orientation. Chain 1 has additional mirror symmetry in the plane of Figure 2b, and is found at higher temperatures than Chain 2. In PipBF_4_–II and −IV, adjacent chains in the [110] direction point antiparallel to one another whereas in the other phases they are parallel (defined by the directionality of upright piperaziniums as indicated by the pink lines in Figure 7b). Generally, the packing of antiparallel chains in PipBF_4_ appears to be more favorable so the selection of parallel chains by PipClO_4_ and PipReO_4_ is likely due to a stabilizing effect of N–H···O hydrogen bonds between the perchlorate/rhenate anions and piperazinium cations which does not occur with BF_4_^–^.^28^

The relationship between PipBF_4_–I and −II is depicted in Figure 8. These two polymorphs are significantly separated on different branches of the subgroup tree, and the transition has to occur via the hypothetical *P*4/*mmm* parent. The structural changes required are larger than those between II to IV, which are close to each other on the subgroup tree. This is consistent with the observed loss of single crystal integrity during the transition. Chains in PipBF_4_–II adopt the Chain 1 configuration and these are related to one another by an *n* glide plane, such that adjacent chains point antiparallel to one another (the reflection in the *ac*-plane reverses the direction of the upright piperaziniums but maintains the dipole direction along the *b*-axis). The noncentrosymmetry of this phase indicated by SHG measurements helped inform the space-group choice of *Pmn*2_1_ over *Pnnm* during SXRD analysis. This symmetry breaking is due to the hydrogen atoms ordering in the hydrogen bonds such that piperazinium dipoles are parallel despite the chains themselves being antiparallel. On cooling, PipBF_4_–II transitions first to polymorph III (384 K) then to IV (346 K). Polymorph IV is related to II by the rotation of the flat piperaziniums to face-on that transforms Chain 1 into Chain 2. This rotation out of the *ac*-plane lengthens the *b*-axis and shortens the *a*-axis significantly. This results in large shifts in the (020) (white arrow) and (200) (light blue arrow) reflections seen on the PXRD surface plot of Figure 3. The β angle changes from 90° to 98.1° (at room temperature) as seen by the splitting of the (112) peak into (112) and (112̅) (yellow arrows) of the monoclinic cell. The single irrep linking the two phases in Figure 8b shows that the transition from II to IV has the potential to be continuous.

In fact, this transition is not continuous as PipBF_4_–III forms between these phases. PipBF_4_–III has a chain configuration that is significantly different as highlighted by the distance between it and the temperature-adjacent phases in the subgroup tree of Figure 8. All the chains now point parallel rather than antiparallel (Figure 7). 2/3 of the chains adopt the Chain 2 configuration with half of these having hydrogen order such that the piperazinium dipoles point along [010] and the other half along [01̅0] to satisfy the centrosymmetry of *P*1̅. The remaining 1/3 of the chains retain the Chain 1 configuration with disordered hydrogen bonds and the whole chain undergoes a 90° rotation about the chain direction. To help clarify the relationship between phases IV and III, the structure of phase III can be transformed onto the unit-cell basis of phase II and IV. The transformation matrix and resulting cell parameters are given in Table 1. This shows that the cell of phase III is a 3 × 3 × 1 supercell of phase IV. Figure 9 shows phases II, III and IV viewed along the *c*-axis highlighting the chain polarization configuration. The nonpolar nature of PipBF_4_–III is clear from the equal number of in and out polar chains. The chain arrangement is clearly different for phase III than for phases IV and II. There are at least four irreps between each of IV or II to III, which is consistent with the two discontinuous transitions observed in the IV→III→II phase sequence and the significant symmetry decrease from the large triclinic cell of phase III to the smaller orthorhombic and monoclinic cells of phases II and IV, respectively.

**Figure 9:**
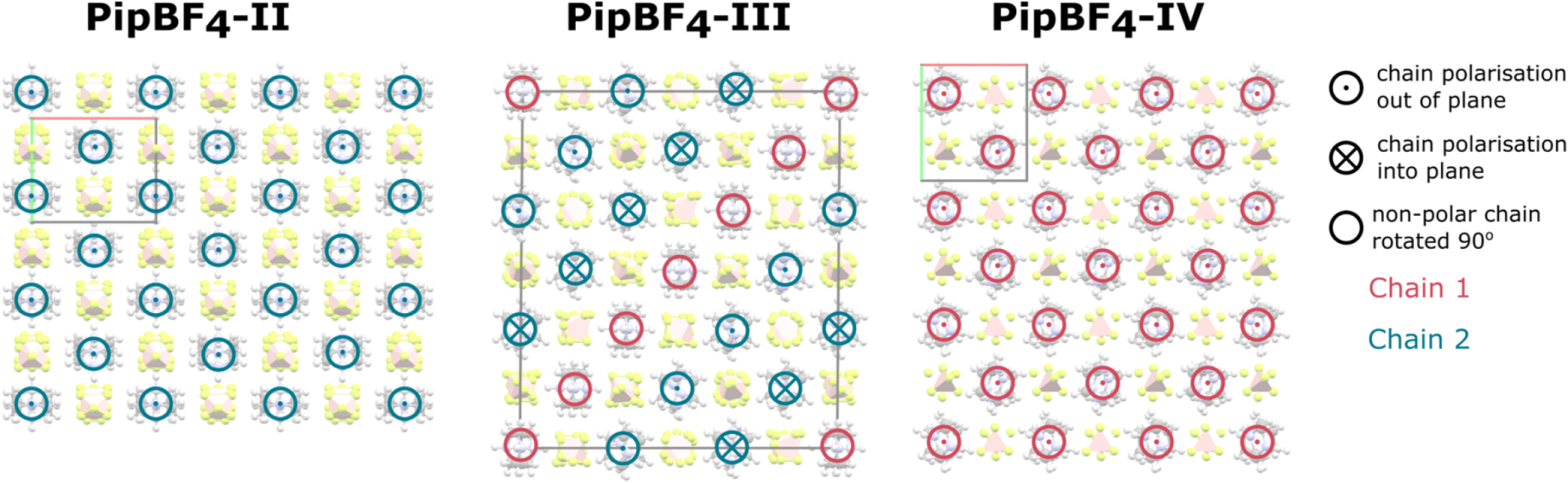
Structures of polymorphs II/III/IV of PipBF_4_ with the III structure transformed onto the IV basis; views are along the *c*-axis of PipBF_4_–II.

The known structures of PipClO_4_ and the new structures of PipReO_4_ can also be rationalized on the same group-subgroup tree. They are reasonably close on the tree to most PipBF_4_ structures, but there is no direct transformation between the PipClO_4_ structures and PipBF_4_–II (i.e., without going through the *Immm* parent). To better visualize the similarities between PipClO_4_–V, −III, PipReO_4_–II, −I and PipBF_4_, the structures were transformed onto the PipBF_4_–II/–IV unit-cell basis. These are visualized in Figure 10 which shows that they have very similar structures to PipBF_4_–IV – all chains are in the Chain 2 configuration – with the key difference being the parallel chains in PipBF_4_ and antiparallel chains in PipClO_4_ and PipReO_4_, as previously discussed.

**Figure 10:**
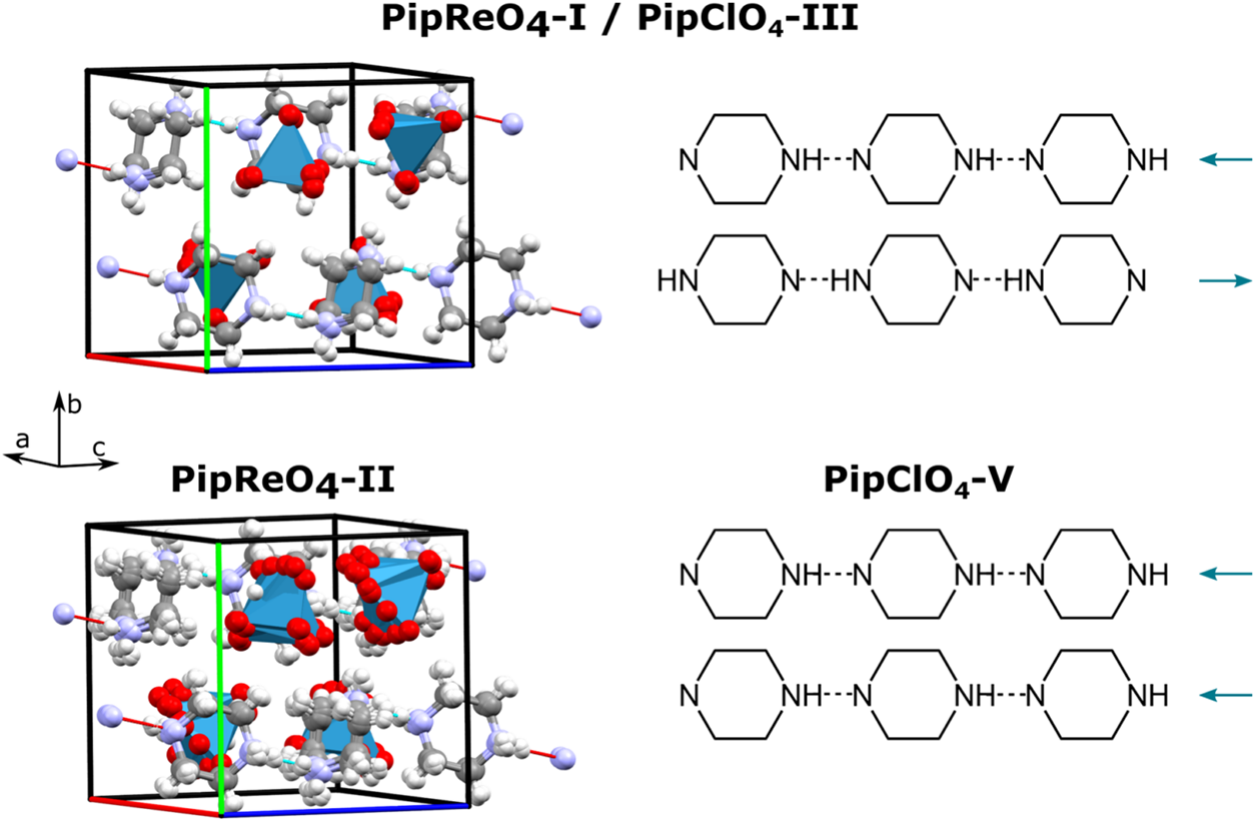
Unit cells of the two new structures of PipReO_4_ transformed onto the PipBF_4_–IV axes (left). Hydrogen ordering with hydrogen-bonded chains of piperazinium in known phases of PipReO_4_ and PipClO_4_ (right).

PipClO_4_–V and PipReO_4_–II can be derived as childs of the isostructural PipClO_4_–III and PipReO_4_–I. This parent structure is made up of parallel hydrogen-bonded chains of piperazinium with the polarization of adjacent chains antiparallel leading to a nonpolar structure. The transition to PipClO_4_–V is driven by the chain polarizations becoming parallel, forming a potentially ferroelectric structure based on switchable hydrogen bonds. The transition to PipReO_4_–II maintains the nonpolar distribution of chains and instead involves slight positional differences in the cations and anions allowed by the higher *Z* of the large triclinic cell.

## 4 Conclusions

This work presents a detailed structural investigation into the molecular salt piperazinium tetrafluoroborate (PipBF_4_), revealing six polymorphs between 120 and 440 K, including two previously unreported polymorphs. Using a combination of single-crystal and powder X-ray diffraction, supported by variable-temperature SHG measurements, we solve all six structures including a *P*2_1_/*n* phase which forms a ferroelectric/paraelectric pair with the room temperature *Pn* structure. The structural transformations are directed by changes in piperazinium orientations, changes in hydrogen-bond ordering and rotational disorder. All these changes can be rationalized using isotropy group–subgroup analysis.

We expanded the structural family by synthesizing the perrhenate analogue (PipReO_4_), which exhibits room and low temperature polymorphs. These, as well as the previously reported polymorphs of piperazinium perchlorate, are included in the subgroup analysis.

More generally, the study highlights the complexity of structural changes that can be supported in “simple” molecular systems, and how they can impact materials properties. The combination of symmetry detection tools such as FERROSCOPE to identify potential phase transitions^7,8^ and symmetry-mode language to understand structural relationships brings significant insight to molecular solids.^29^

## Supplementary Material



## Notes

Single-crystal X-ray diffraction structures and data; powder X-ray diffraction data and Rietveld information; Rietveld plots for PipReO_4_; thermal expansion plot for PipReO_4_; SHG setup details; unit cells of solved crystal structures; experimental details and refinement data for additional crystal structure determinations (PDF)
