# Supplementary material for: Multiple Polymorphs and their Relationships in the Potential Ferroelectric Piperazinium Tetrafluoroborate
Source: Cryst Growth Des. 2025 Dec 5;25(24):10461–72. doi: 10.1021/acs.cgd.5c01210 (PMC12715757; doi:10.1021/acs.cgd.5c01210)
Supplement: Supplementary file 1 [file cg5c01210_si_001.pdf]

Multiple polymorphs and their relationships in the potential ferroelectric piperazinium tetrafluoroborate

Sam Y. Thompson, Ella I. D. Thomson, Faith G. Pritchard, Toby J. Blundell, Jan R. R. Verlet and John S. O. Evans*

Department of Chemistry, Durham University, Lower Mount Joy, South Road, Durham, DH1 3LE, United Kingdom

Supplementary Information

PipReO4

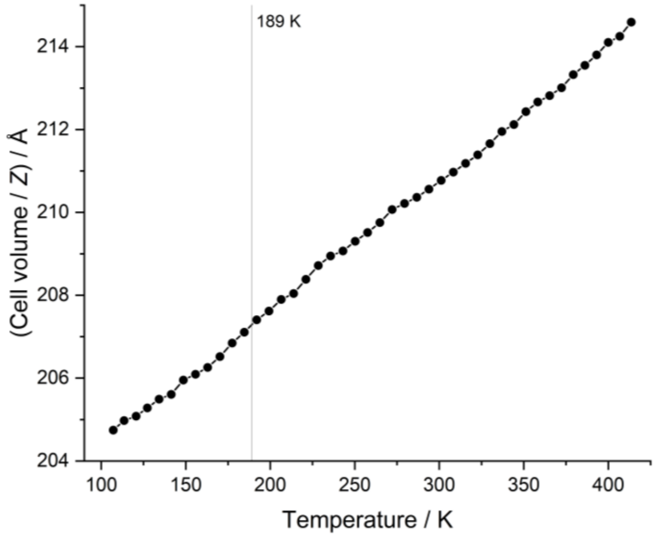

Figure S1. Thermal expansion of $PipReO_4$.

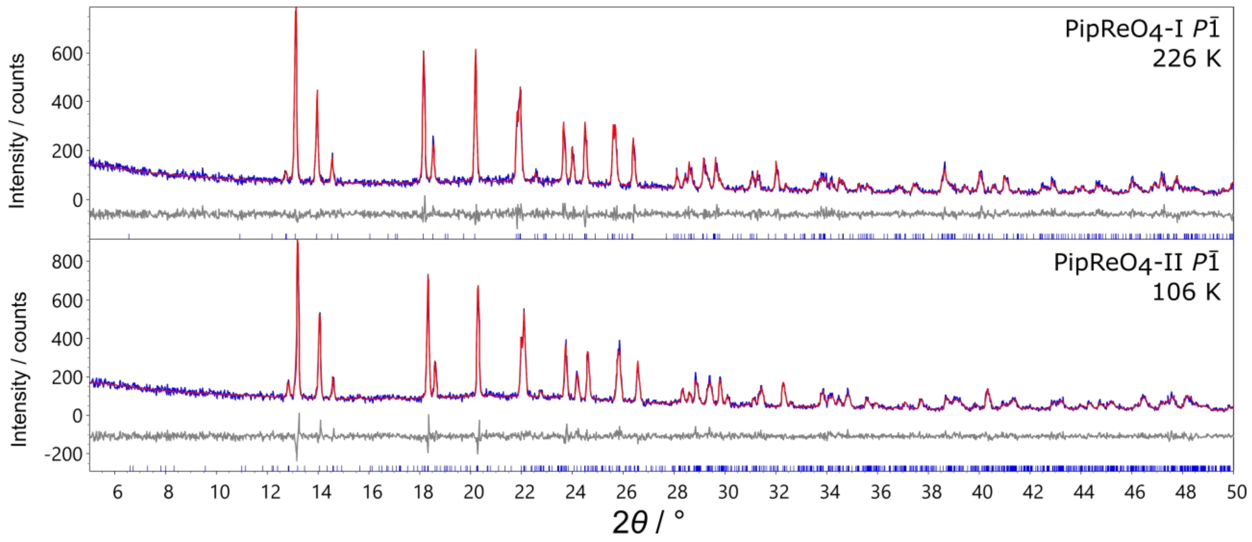

Figure S2. Powder X-ray diffraction data (blue) of $PipReO_4$ showing the calculated pattern from the Rietveld refinements (red) and the difference curves (gray).

Unit cells

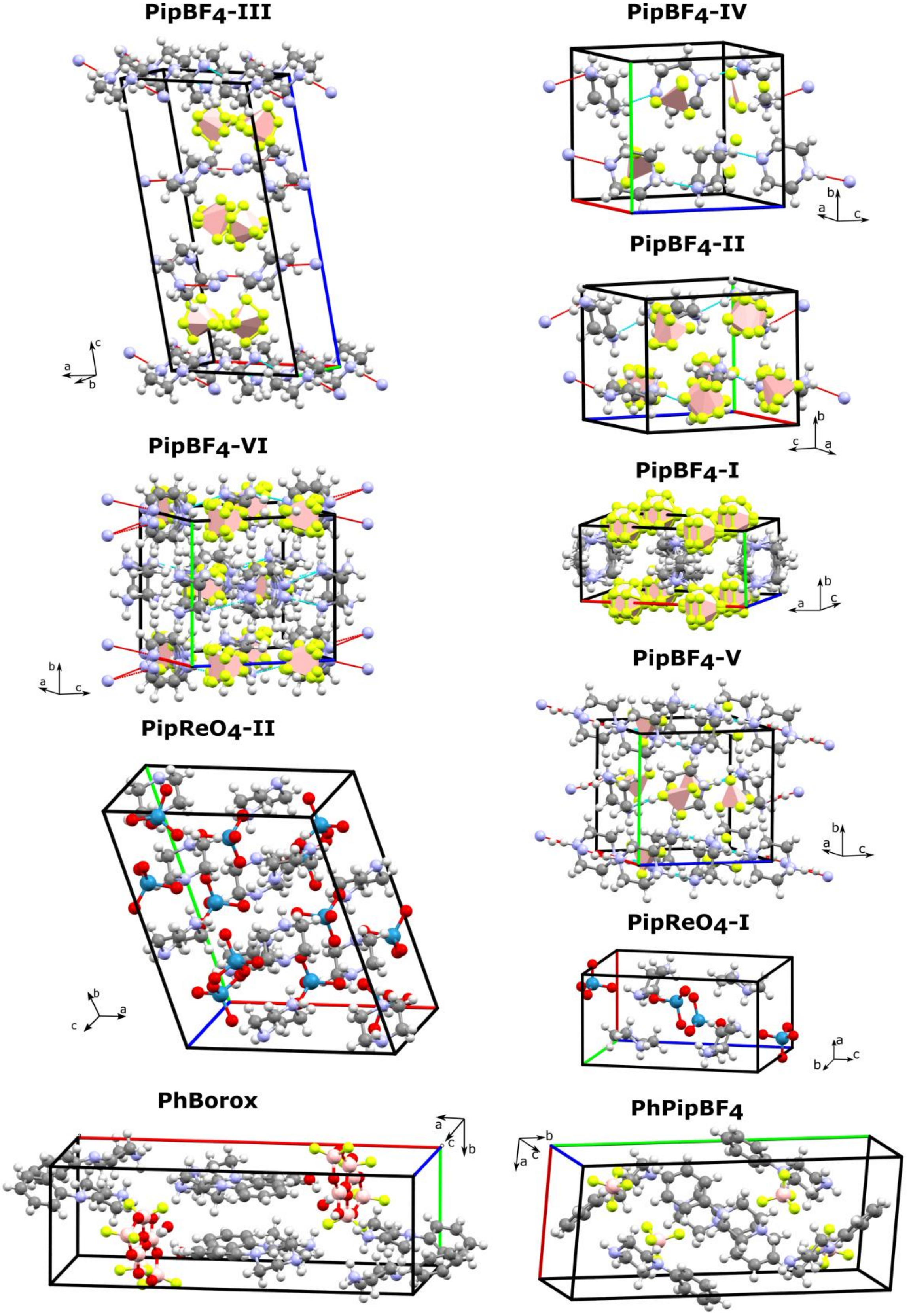

Figure S3. A single unit cell of each of the new structures reported.

SHG generation

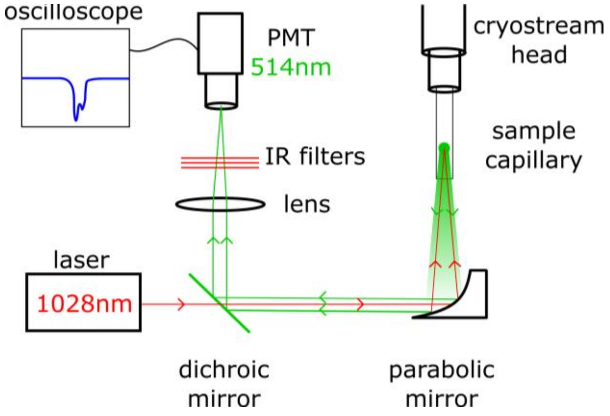

Figure S4. Schematic of experimental setup for variable-temperature second-harmonic generation.

Additional crystal structures

Two other crystal structures were observed during the course of this investigation which are not discussed in the main text. Stoichiometric amounts of 1-phenyl piperazine (0.46 ml, 3 mmol) and tetrafluoroboric acid (0.20 ml, 3 mmol) were combined in methanol (5.0 ml) and stirred for 10 minutes. The solution was filtered and left to evaporate overnight until plate-like colourless crystals were obtained. 1-phenylpiperazine 4-phenyl-1-piperazinium tetrafluoroborate ($PhPipBF_4$) was the main product. A minor side product (PhBorox) was produced which features 4-phenyl-1-piperazinium ion and a new fluoroboroxinol dianion. We could not find any evidence of structures in Cambridge Structural Database that feature this ion. $PhPipBF_4$ and PhBorox have CCDC codes 2472723 and 2472724, respectively.

$PipBF_4$ refinements

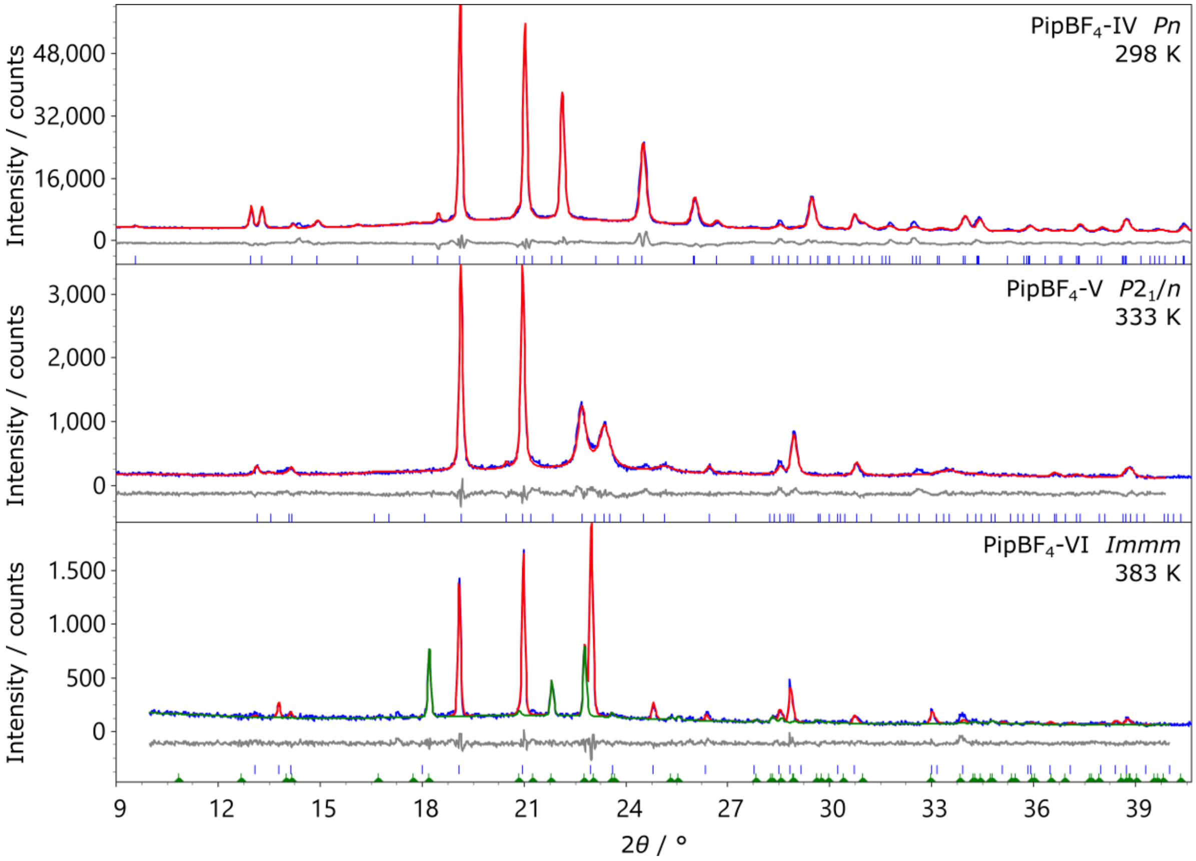

Figure S6. Rietveld refinements reproduced from Figures 5 and 6 to aid in comparison across structures.

Refinement tables

Table S1. – Crystal data and Rietveld refinement information for $PipBF_4$-I.

Empirical formula	$C_4N_2H_{11}$, BF_4	Radiation	Cu Kα
Formula weight	173.95	2θ / °	3 - 60
Space group	*Pmma*	T / K	415
Unit cell dimensions		B_{eq} / Å^2	
a / Å	12.3084(9)	$C_2N_4H_{11}$	14.3(7)
b / Å	5.2959(4)	BF_4	18.7(10)
c / Å	6.2734(6)	R_{wp} (%)	5.18
V / Å^3	408.93(6)	R_{Bragg} (%)	1.95
Z	2	R_p (%)	3.81
D_x / g cm^{-3}	1.41271(19)	GOF	2.81

Table S2. Crystal data and Rietveld refinement information for $PipBF_4$-II.

Empirical formula	$C_4N_2H_{11}$, BF_4	Radiation	Cu Kα
Formula weight	173.95	2θ / °	3 - 60
Space group	$Pmn2_1$	T / K	394
Unit cell dimensions		B_{eq} / Å^2	8.2(2)
a / Å	9.7273(8)	R_{wp} (%)	4.44
b / Å	8.1326(6)	R_{Bragg} (%)	3.84
c / Å	10.0028(6)	R_p (%)	3.11
V / Å^3	791.30(10)	GOF	4.30
Z	4		
D_x / g cm^{-3}	1.46435(18)		

Table S3. Crystal data and Rietveld refinement information for $PipBF_4$-III.

Empirical formula	$C_4N_2H_{11}$, BF_4	Radiation	Cu Kα
Formula weight	173.95	2θ / °	3 - 60
Space group	$P\bar{1}$	T / K	357
Unit cell dimensions		B_{eq} / Å^2	10.7(3)
a / Å	7.9815(6)	R_{wp} (%)	3.27
b / Å	8.0185(7)	R_{Bragg} (%)	2.27
c / Å	19.0955(16)	R_p (%)	2.32
α / °	81.331(2)	GOF	5.11
β / °	79.043(2)		
γ / °	76.249(3)		
V / Å^3	1158.28(17)		
Z	6		
D_x / g cm^{-3}	1.4963(2)		

Table S4. Crystal data and Rietveld refinement information for $PipBF_4$-IV.

Empirical formula	$C_4N_2H_{11}$, BF_4	Radiation	Cu Kα
Formula weight	173.95	2θ / °	10 – 50
Space group	Pn	T / K	298
Unit cell dimensions		B_{eq} / Å^2	7.5(3)
a / Å	8.4751(7)	R_{wp} (%)	5.63
b / Å	9.2215(8)	R_{Bragg} (%)	3.12
c / Å	9.6396(7)	R_p (%)	3.99
β / °	98.089(4)	GOF	3.52
V / Å^3	745.87(11)		
Z	4		
D_x / g cm^{-3}	1.5490(2)		

Table S5. Crystal data and Rietveld refinement information for $PipBF_4$-V.

Empirical formula	$C_4N_2H_{11}$, BF_4	Radiation	Cu Kα
Formula weight	173.95	2θ / °	3 - 60
Space group	$P2_1/n$	T / K	333
Unit cell dimensions		B_{eq} / Å^2	1.8(9)
a / Å	8.449(2)	R_{wp} (%)	8.45
b / Å	9.235(3)	R_{Bragg} (%)	1.76
c / Å	9.7842(19)	R_p (%)	6.49
β / °	92.276(14)	GOF	1.37
V / Å^3	762.8(3)		
Z	4		
D_x / g cm^{-3}	1.5147(6)		

Table S6. Crystal data and Rietveld refinement information for $PipBF_4$-VI.

Empirical formula	$C_4N_2H_{11}$, BF_4	Radiation	Cu Kα
Formula weight	173.95	2θ / °	10 - 50
Space group	$Immm$	T / K	383
Unit cell dimensions		B_{eq} / Å^2	0.10 ± 1.34
a / Å	8.4621(16)	R_{wp} (%)	10.53
b / Å	9.2890(19)	R_{Bragg} (%)	2.63
c / Å	9.8423(15)	R_p (%)	8.11
V / Å^3	773.6(2)	GOF	1.27
Z	4		
D_x / g cm^{-3}	1.4761(5)		

Table S7. Crystal data and Rietveld refinement information for $PipReO_4$-I.

Empirical formula	$C_4N_2H_{11}$, ReO_4	Radiation	Cu Kα
Formula weight	337.35	2θ / °	3 - 60
Space group	$P\bar{1}$	T / K	226
Unit cell dimensions		B_{eq} / Å^2	
a / Å	7.6258(5)	$C_4N_2H_{11}$	8.4(11)
b / Å	8.4328(6)	ReO_4	6.2(3)
c / Å	13.6224(10)	R_{wp} (%)	13.68
α / °	88.196(4)	R_{Bragg} (%)	5.06
β / °	82.638(4)	R_p (%)	10.59
γ / °	73.890(4)	GOF	1.15
V / Å^3	834.67(10)		
Z	4		
D_x / g cm^{-3}	2.6846(3)		

Table S8. Crystal data and Rietveld refinement information for $PipReO_4$-II.

Empirical formula	$C_4N_2H_{11}$, ReO_4	Radiation	Cu Kα
Formula weight	337.35	2θ / °	3 - 60
Space group	$P\bar{1}$	T / K	106
Unit cell dimensions		B_{eq} / Å^2	4.3(3)
a / Å	12.7380(8)	R_{wp} (%)	12.04
b / Å	15.1689(9)	R_{Bragg} (%)	4.43
c / Å	15.6813(10)	R_p (%)	9.33
α / °	103.788(4)	GOF	1.12
β / °	110.931(4)		
γ / °	108.816(4)		
V / Å^3	2455.3(3)		
Z	12		
D_x / g cm^{-3}	2.7379(3)		

Table S9. Crystal data and structure refinement details for $PipBF_4$-IV.

Identification code	PipBF4-IV
Empirical formula	$C_4H_{11}BF_4N_2$
Formula weight	173.96
Temperature/K	120.0(2)
Crystal system	monoclinic
Space group	*Pn*

a/Å	8.3950(5)
b/Å	9.1069(6)
c/Å	9.4858(6)
α/°	90
β/°	98.852(2)
γ/°	90
Volume/Å^3	716.57(8)
Z	4
ρ_{calc}g/cm^3	1.612
μ/mm^{-1}	0.170
F(000)	360.0
Crystal size/mm^3	0.237 × 0.158 × 0.057
Radiation	MoKα (λ = 0.71073)
2Θ range for data collection/°	4.472 to 63.102
Index ranges	-12 ≤ h ≤ 12, -13 ≤ k ≤ 13, -13 ≤ l ≤ 13
Reflections collected	16458
Independent reflections	4757 [R_{int} = 0.0350, R_{sigma} = 0.0384]
Data/restraints/parameters	4757/22/301
Goodness-of-fit on F^2	1.030
Final R indexes [I>=2σ (I)]	R_1 = 0.0342, wR_2 = 0.0710
Final R indexes [all data]	R_1 = 0.0426, wR_2 = 0.0752
Largest diff. peak/hole / e Å^{-3}	0.32/-0.20
Flack parameter	0.3(2)

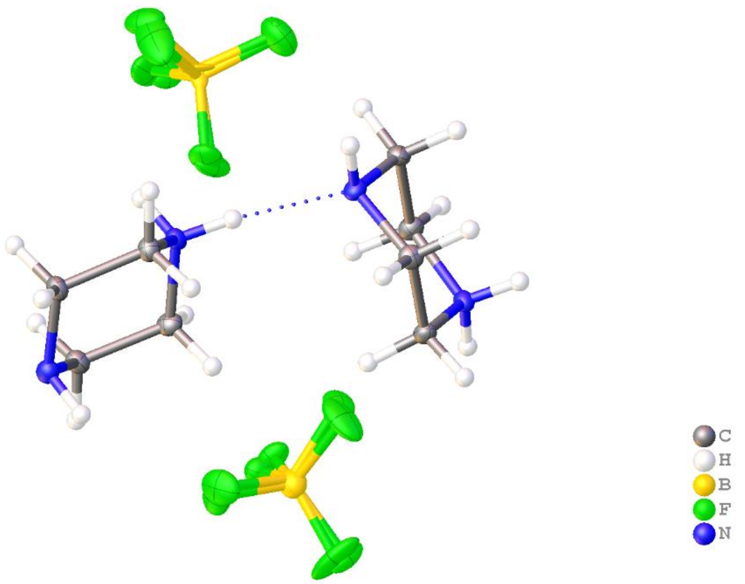

Table S10. Fractional Atomic Coordinates (×10^4) and Equivalent Isotropic Displacement Parameters (Å^2×10^3) for PipBF$_4$-IV. U_{eq} is defined as 1/3 of the trace of the orthogonalised U_{IJ} tensor.

Atom	x	y	z	U(eq)
F1	5370(20)	8340(20)	4900(20)	20(2)
F2	7959(19)	8272(18)	5840(30)	43(4)
F3	6250(20)	6259(17)	6094(17)	25(2)
F4	7100(40)	6820(40)	4010(30)	31(4)
B1	6680(20)	7390(20)	5260(20)	7(4)
F5	20(20)	3574(14)	3971(19)	22(2)
F6	1074(12)	2080(20)	5753(12)	16(2)
F7	2690(20)	3110(30)	4280(20)	23(3)
F8	892(12)	1302(14)	3474(15)	30.2(19)
B2	1160(20)	2470(20)	4360(20)	17(4)
N1	6836(3)	3806(2)	8089(2)	12.8(4)
N2	6102(3)	1266(2)	6318(2)	13.3(4)
C1	5149(3)	3456(2)	7418(3)	14.6(4)

Table S10. Fractional Atomic Coordinates (×10⁴) and Equivalent Isotropic Displacement Parameters (Å²×10³) for PipBF$_4$-IV. U_{eq} is defined as 1/3 of the trace of the orthogonalised U_{IJ} tensor.

Atom	x	y	z	U(eq)
C2	8052(3)	3174(3)	7259(3)	14.0(4)
C3	4944(3)	1822(2)	7217(3)	13.8(4)
C4	7759(3)	1533(2)	7043(3)	14.7(4)
N3	5627(3)	2014(2)	3382(2)	12.8(3)
N4	7232(3)	2968(2)	1022(2)	14.3(4)
C5	6098(3)	3583(2)	3231(3)	15.1(4)
C6	6746(3)	1002(2)	2760(3)	13.7(4)
C7	6167(3)	3954(2)	1679(2)	14.6(4)
C8	6816(3)	1410(2)	1214(3)	14.3(4)
B2A	1290(20)	2550(17)	4353(12)	12(3)
F5A	6(16)	3390(20)	3680(20)	34(3)
F6A	1077(15)	2190(20)	5717(13)	37(3)
F7A	2699(16)	3340(20)	4400(20)	25(3)
F8A	1420(50)	1275(11)	3573(13)	55(4)
F1A	5271(14)	8152(17)	5151(17)	20.1(17)
F2A	7947(11)	8322(9)	6021(13)	23.3(15)
F3A	6730(30)	6173(10)	6092(11)	40(2)
F4A	7080(20)	7040(20)	3946(14)	27(2)
B1A	6751(18)	7444(16)	5276(16)	20(4)

Table S11. Crystal data and structure refinement details for PipBF$_4$-III.

Identification code	PipBF4-III
Empirical formula	$C_4H_{11}BF_4N_2$
Formula weight	173.96
Temperature/K	370.0(2)
Crystal system	triclinic
Space group	$P\bar{1}$
a/Å	8.0065(5)
b/Å	8.0351(6)
c/Å	19.1239(12)
α/°	81.381(5)
β/°	79.057(5)
γ/°	76.226(6)
Volume/Å³	1166.10(14)
Z	6
ρ_{calc}g/cm³	1.486
μ/mm⁻¹	1.413
F(000)	540.0

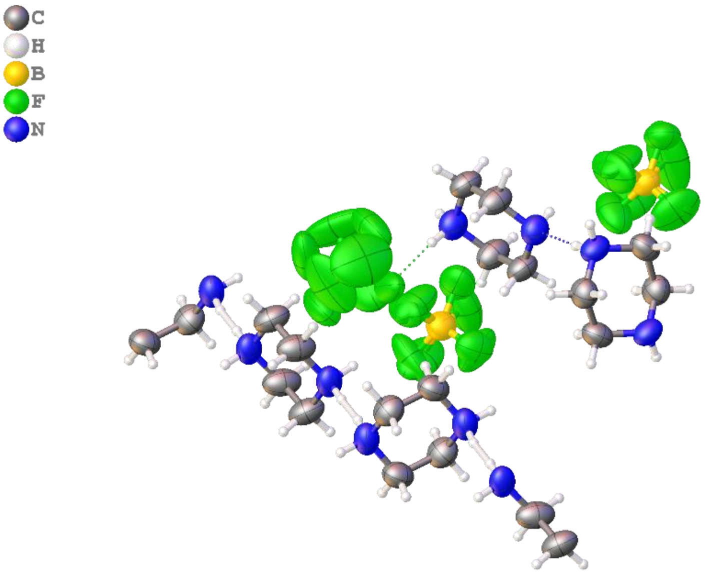

Crystal size/mm^3	0.484 × 0.202 × 0.07
Radiation	MoKα (λ = 1.54184)
2Θ range for data collection/°	4.734 to 149.312
Index ranges	-9 ≤ h ≤ 9, -10 ≤ k ≤ 9, -23 ≤ l ≤ 23
Reflections collected	9022
Independent reflections	9022 [R_{int} = ?, R_{sigma} = 0.0694]
Data/restraints/parameters	9022/750/442
Goodness-of-fit on F^2	0.794
Final R indexes [I>=2σ (I)]	R_1 = 0.0925, wR_2 = 0.2282
Final R indexes [all data]	R_1 = 0.1425, wR_2 = 0.2485
Largest diff. peak/hole / e Å^{-3}	0.36/-0.43

Table S12. Fractional Atomic Coordinates (×10^4) and Equivalent Isotropic Displacement Parameters (Å^2×10^3) for PipBF$_4$-III. U_{eq} is defined as 1/3 of the trace of the orthogonalised U_{IJ} tensor.

Atom	x	y	z	U(eq)
N1	8138(4)	6641(4)	3751.9(17)	70.5(9)
N2	8162(4)	9931(4)	2969.0(18)	72.6(9)
C1	7372(5)	7147(5)	3086(2)	74.2(10)
C2	8292(5)	8406(5)	2601.2(19)	75.8(11)
C3	9047(5)	9403(5)	3581(2)	77.8(11)
C4	8151(5)	8165(5)	4098.6(19)	75.8(11)
N3	11635(4)	4574(4)	3569.8(19)	76.8(9)
N4	14680(4)	2072(4)	3056.9(19)	77.1(9)
C5	11683(5)	2756(5)	3703(2)	82.4(12)
C6	13487(5)	1615(5)	3705(2)	76.9(11)
C7	14672(5)	3900(5)	2922(3)	85.1(13)
C8	12832(5)	4993(4)	2948(2)	76.4(11)
N5	15568(5)	3627(4)	-429.8(19)	79.1(10)
C9	14186(6)	4659(5)	715(2)	79.8(12)
C10	14016(5)	3700(5)	118(2)	79.6(11)
N6	18613(4)	1511(4)	53.7(18)	75.0(9)
C11	18283(5)	-212(6)	301(2)	91.0(14)
C12	19901(5)	-1463(5)	513(2)	87.1(13)
B1	13606(7)	-431(6)	1796(3)	77.7(12)
F1	12476(15)	-681(19)	1472(7)	221(6)
F2	13026(18)	-477(15)	2495(4)	153(4)
F3	14630(18)	638(17)	1676(9)	196(7)
F4	14795(12)	-1906(12)	1676(6)	165(4)
F1A	11930(12)	-260(20)	1993(10)	249(7)
F2A	13880(20)	1214(12)	1781(6)	177(6)

Table S12. Fractional Atomic Coordinates (×10⁴) and Equivalent Isotropic Displacement Parameters (Å²×10³) for PipBF$_4$-III. U_{eq} is defined as 1/3 of the trace of the orthogonalised U_{IJ} tensor.

Atom	x	y	z	U(eq)
F3A	14370(20)	-1430(18)	2278(10)	244(8)
F4A	14041(18)	-439(12)	1091(4)	177(4)
B2	12956(6)	7175(5)	4928(3)	72.5(12)
F5	12058(14)	5896(12)	5040(6)	137(4)
F6	13963(17)	6843(19)	4285(6)	145(5)
F7	13781(18)	7400(20)	5409(9)	277(9)
F8	11710(20)	8514(18)	4763(10)	167(7)
F5A	11881(18)	8769(14)	4863(6)	114(4)
F6A	12985(11)	6767(9)	5662(4)	103(2)
F7A	14505(13)	7151(16)	4550(7)	175(6)
F8A	12170(20)	5933(17)	4856(7)	177(6)
B3	18938(7)	3913(6)	1548(3)	79.7(13)
F9	19214(17)	3572(13)	2240(5)	129(4)
F10	17508(17)	5278(17)	1492(6)	106(3)
F11	20129(18)	3998(19)	967(7)	171(7)
F12	18280(20)	2513(16)	1527(7)	150(6)
F9A	19913(17)	4256(18)	1966(7)	182(6)
F10A	20020(20)	3690(20)	899(6)	150(6)
F11A	17870(20)	5441(15)	1471(10)	149(6)
F12A	18092(15)	2602(12)	1666(8)	133(5)

Table S13. Crystal data and structure refinement details for PipBF$_4$-II.

Identification code	PipBF4-II
Empirical formula	$C_4H_{11}BF_4N_2$
Formula weight	173.96
Temperature/K	385.0(2)
Crystal system	orthorhombic
Space group	*Pmn*2$_1$
a/Å	9.7292(8)
b/Å	8.1355(7)
c/Å	9.9939(8)
α/°	90
β/°	90
γ/°	90
Volume/Å^3	791.04(11)
Z	4
ρ_{calc}g/cm^3	1.461
μ/mm^{-1}	0.154

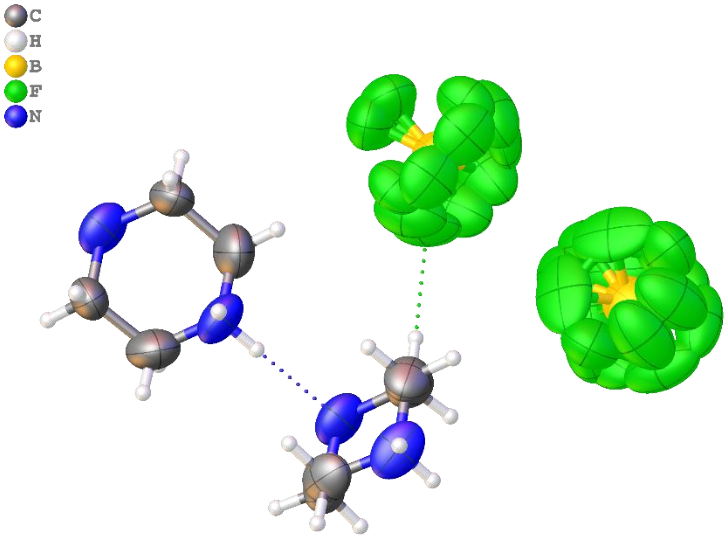

F(000)	360.0
Crystal size/mm^3	0.211 × 0.133 × 0.109
Radiation	MoKα (λ = 0.71073)
2Θ range for data collection/°	5.008 to 51.996
Index ranges	-12 ≤ h ≤ 12, -10 ≤ k ≤ 10, -12 ≤ l ≤ 12
Reflections collected	12417
Independent reflections	1648 [R_{int} = 0.0482, R_{sigma} = 0.0329]
Data/restraints/parameters	1648/809/214
Goodness-of-fit on F^2	1.054
Final R indexes [I>=2σ (I)]	R_1 = 0.0860, wR_2 = 0.2480
Final R indexes [all data]	R_1 = 0.1259, wR_2 = 0.2914
Largest diff. peak/hole / e Å^{-3}	0.35/-0.23
Flack parameter	0.0(7)

Table S14. Fractional Atomic Coordinates (×10^4) and Equivalent Isotropic Displacement Parameters (Å^2×10^3) for PipBF$_4$-II. U_{eq} is defined as 1/3 of the trace of the orthogonalised U_{IJ} tensor.

Atom	x	y	z	U(eq)
N1	0	8806(11)	-320(11)	91(3)
N4	0	7482(11)	-7674(10)	81(3)
C1	1230(8)	8414(12)	-1122(10)	88(3)
N2	0	6165(11)	-2149(10)	79(3)
C3	1192(9)	7305(14)	-5526(10)	100(4)
N3	0	7667(12)	-4821(9)	81(3)
C2	1256(8)	6639(12)	-1418(10)	85(2)
C4	1256(10)	7909(15)	-6937(9)	92(3)
B1	5000	7046(11)	-3725(8)	81(3)
F2	5420(30)	8490(20)	-3150(30)	108(10)
F1	5000	7260(30)	-5099(11)	116(8)
F4	5990(20)	5810(20)	-3490(30)	83(8)
F3	3780(20)	6340(30)	-3340(40)	117(11)
B2	5000	7605(11)	990(9)	78(3)
F8	6030(30)	8130(60)	150(40)	147(11)
F6	4020(20)	8860(20)	940(40)	107(8)
F5	4280(40)	6300(30)	440(40)	140(12)
F7	5480(30)	7370(40)	2273(18)	118(9)
F1A	4550(30)	5860(30)	-2860(30)	123(10)
F3A	4030(20)	8320(20)	-3600(30)	83(8)
F4A	5000	6630(40)	-5057(14)	136(10)
F2A	6230(20)	7760(40)	-3340(40)	105(10)
F7A	5000	8010(40)	-341(14)	181(11)
F5A	6060(20)	6440(30)	970(30)	92(7)

Table S14. Fractional Atomic Coordinates (×10^4) and Equivalent Isotropic Displacement Parameters (Å^2×10^3) for PipBF$_4$-II. U$_{eq}$ is defined as 1/3 of the trace of the orthogonalised U$_{IJ}$ tensor.

Atom	x	y	z	U(eq)
F8A	3840(30)	6860(40)	1490(40)	125(10)
F6A	5530(30)	8870(30)	1730(30)	141(10)

Table S15. Crystal data and structure refinement details for PipReO$_4$-I.

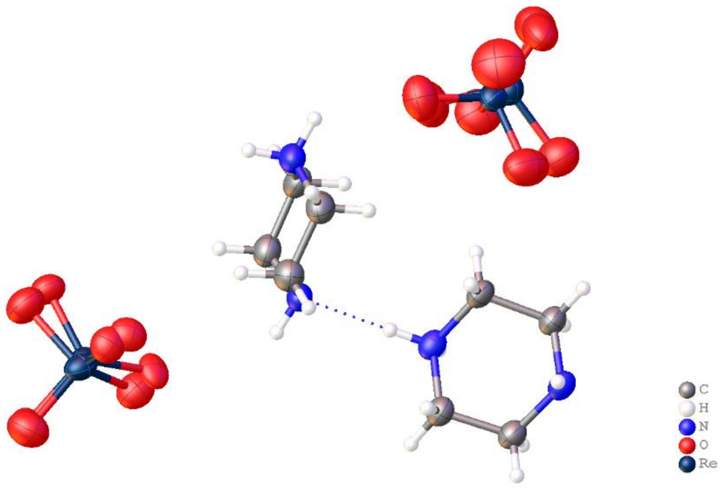

Identification code	PipReO4-I
Empirical formula	$C_4H_{11}N_2O_4Re$
Formula weight	337.350
Temperature/K	298.0(2)
Crystal system	triclinic
Space group	$P\bar{1}$
a/Å	7.6478(8)
b/Å	8.4670(8)
c/Å	13.6549(13)
α/°	88.233(4)
β/°	82.646(4)
γ/°	73.807(4)
Volume/Å^3	842.13(14)
Z	4
ρ_{calc}g/cm^3	2.661
μ/mm^{-1}	14.410
F(000)	622.2
Crystal size/mm^3	0.149 × 0.115 × 0.109
Radiation	MoKα (λ = 0.71073)
2Θ range for data collection/°	5 to 60
Index ranges	$-11 \le h \le 11$, $-12 \le k \le 12$, $-20 \le l \le 20$
Reflections collected	23170
Independent reflections	4890 [R_{int} = 0.0347, R_{sigma} = 0.0304]
Data/restraints/parameters	4890/528/298
Goodness-of-fit on F^2	1.029
Final R indexes [I>=2σ (I)]	R_1 = 0.0521, wR_2 = 0.1306
Final R indexes [all data]	R_1 = 0.0635, wR_2 = 0.1353
Largest diff. peak/hole / e Å^{-3}	2.57/-2.77

Table S16. Fractional Atomic Coordinates (×10^4) and Equivalent Isotropic Displacement Parameters (Å^2×10^3) for PipReO$_4$-I. U$_{eq}$ is defined as 1/3 of the trace of the orthogonalised U$_{IJ}$ tensor.

Atom	X	y	z	U(eq)
Re1	3348(10)	2420(8)	5079(5)	32.3(6)

Table S16. Fractional Atomic Coordinates (×10[4]) and Equivalent Isotropic Displacement Parameters (Å[2]×10[3]) for PipReO$_4$-I. U_{eq} is defined as 1/3 of the trace of the orthogonalised U_{IJ} tensor.

Atom	X	y	z	U(eq)
O1	2180(30)	1800(20)	4238(17)	60(5)
O2	5680(30)	1700(30)	4818(13)	46(4)
O4	2640(40)	1880(30)	6269(17)	52(5)
O3	2910(30)	4480(20)	5017(14)	49(4)
Re1a	11529(6)	2617(3)	9963.0(18)	39.5(6)
O8a	12120(30)	3230(30)	11003(15)	74(5)
O6a	11850(40)	590(30)	10024(16)	83(5)
O7a	9350(30)	3230(30)	9933(15)	72(5)
O5a	12410(40)	3030(30)	8845(16)	84(6)
Re2	12041(6)	2730(4)	9956(3)	47.5(5)
O8	10680(40)	2830(40)	10940(20)	101(6)
O5	13310(30)	3980(30)	10011(19)	78(6)
O7	10780(40)	3560(30)	8990(20)	89(6)
O6	13360(30)	780(30)	9675(18)	74(5)
Re2a	3116(13)	2234(11)	5125(5)	40.0(11)
O1a	1570(40)	1390(30)	4703(19)	69(6)
O2a	5370(30)	1550(30)	4516(19)	57(5)
O3a	2160(40)	4300(30)	4974(19)	64(5)
O4a	3200(40)	1910(30)	6369(16)	52(5)
N1	13771(12)	-3903(10)	7988(7)	37.8(17)
N2	11578(12)	-1203(10)	6927(6)	35.7(16)
C4	12232(15)	-897(12)	7864(8)	39(2)
C1	13054(14)	-4165(12)	7076(8)	39(2)
C3	13993(15)	-2240(12)	8012(8)	41(2)
C2	11321(14)	-2880(12)	6881(8)	40(2)
N3	8005(12)	1096(11)	6891(7)	40.3(18)
N4	7237(13)	3827(11)	8200(7)	44.3(19)
C6	8433(15)	3742(14)	7242(9)	47(2)
C5	7904(15)	2734(14)	6512(8)	44(2)
C8	7195(17)	2159(14)	8573(9)	47(2)
C7	6706(16)	1207(15)	7785(9)	48(2)

Table S17. Crystal data and structure refinement details for PipReO$_4$-II.

Identification code	PipReO4-II
Empirical formula	$C_4H_{11}N_2O_4Re$
Formula weight	337.350
Temperature/K	120.0(2)
Crystal system	triclinic

Space group	$P\bar{1}$
a/Å	12.7437(8)
b/Å	15.1771(9)
c/Å	15.6866(9)
α/°	103.781(2)
β/°	110.959(2)
γ/°	108.814(2)
Volume/Å^3	2458.0(3)
Z	12
ρ_{calc}g/cm^3	2.735
μ/mm^{-1}	14.803
F(000)	1872.0
Crystal size/mm^3	0.57 × 0.143 × 0.135
Radiation	MoKα (λ = 0.71073)
2Θ range for data collection/°	3.59 to 63.528
Index ranges	-18 ≤ h ≤ 18, -22 ≤ k ≤ 22, -23 ≤ l ≤ 23
Reflections collected	70026
Independent reflections	16572 [R_{int} = 0.0395, R_{sigma} = 0.0339]
Data/restraints/parameters	16572/0/622
Goodness-of-fit on F^2	1.038
Final R indexes [I>=2σ (I)]	R_1 = 0.0265, wR_2 = 0.0582
Final R indexes [all data]	R_1 = 0.0327, wR_2 = 0.0607
Largest diff. peak/hole / e Å^{-3}	4.21/-3.04

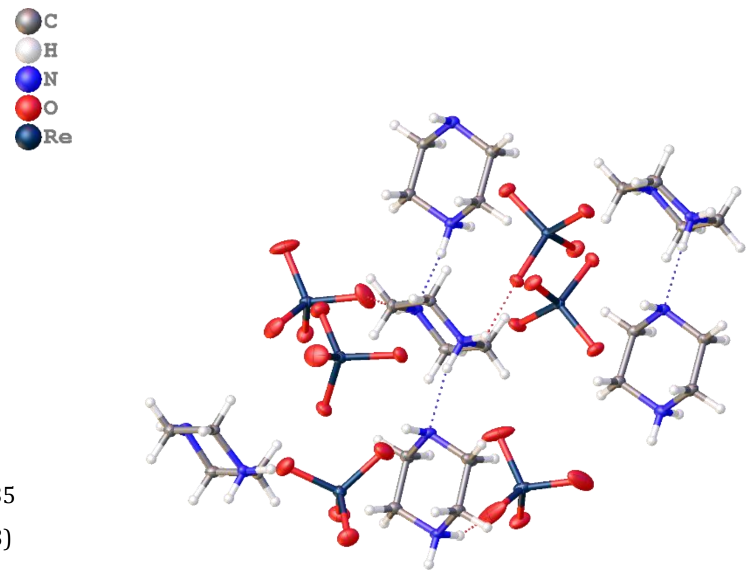

Table S18. Fractional Atomic Coordinates (×10^4) and Equivalent Isotropic Displacement Parameters (Å^2×10^3) for PipReO$_4$-II. U$_{eq}$ is defined as 1/3 of the trace of the orthogonalised U$_{IJ}$ tensor.

Atom	x	y	z	U(eq)
Re1	14314.1(2)	8054.2(2)	5188.4(2)	15.91(3)
O1	14208(3)	6901(2)	4551(2)	35.4(8)
O2	14945(3)	8353(2)	6448(2)	26.0(6)
O3	12855(3)	8042(3)	4771(3)	42.2(9)
O4	15283(4)	8985(3)	5000(3)	45.0(10)
Re2	8868.9(2)	8656.4(2)	4946.4(2)	12.21(3)
O5	7599(3)	7847(2)	4991(2)	22.5(6)
O6	8771(3)	8118(2)	3801(2)	23.3(6)
O7	10215(3)	8816(2)	5903(2)	27.3(6)
O8	8817(3)	9818.0(19)	5115(2)	19.0(5)
Re3	6152.9(2)	6266.4(2)	15.4(2)	12.42(3)
O9	7466(3)	7002(2)	-28(2)	22.4(6)
O10	4827(3)	6071(2)	-997(2)	22.6(6)

Table S18. Fractional Atomic Coordinates (×10^4) and Equivalent Isotropic Displacement Parameters (Å^2×10^3) for PipReO$_4$-II. U$_{eq}$ is defined as 1/3 of the trace of the orthogonalised U$_{IJ}$ tensor.

Atom	x	y	z	U(eq)
O11	6236(3)	6887(2)	1128(2)	24.4(6)
O12	6181(2)	5109.8(19)	-51(2)	18.0(5)
Re4	7586.9(2)	4789.0(2)	5112.8(2)	19.10(3)
O13	8585(4)	5704(3)	4928(2)	47.2(10)
O14	6155(3)	4821(3)	4705(3)	52.7(11)
O15	7425(4)	3629(3)	4463(3)	65.8(15)
O16	8218(3)	5062(2)	6361(2)	31.4(7)
Re5	9085.3(2)	3029.5(2)	91.7(2)	15.85(3)
O17	7750(2)	3182(2)	-36(2)	22.5(6)
O18	10261(3)	4176(2)	367(2)	32.7(7)
O19	8747(4)	2139(3)	-1003(2)	42.6(9)
O20	9551(3)	2650(2)	1061(2)	24.4(6)
Re6	7517.5(2)	212.2(2)	105.6(2)	15.90(3)
O21	8682(3)	295(2)	-239(2)	33.0(7)
O22	8250(3)	741(2)	1390(2)	28.7(7)
O23	6719(3)	854(3)	-358(3)	39.4(8)
O24	6490(3)	-1044(2)	-310(2)	32.7(7)
N1	4016(3)	5248(2)	3042(2)	16.4(6)
N2	4332(3)	6553(2)	1965(2)	14.4(6)
C1	5241(3)	5729(3)	3042(3)	15.6(6)
C2	5390(3)	6702(3)	2875(3)	15.3(6)
C3	3135(3)	6056(3)	1964(3)	16.2(7)
C4	2929(3)	5070(3)	2112(3)	17.2(7)
N3	4630(3)	8384(2)	1723(2)	18.0(6)
N4	7000(3)	10045(2)	3225(2)	16.8(6)
C5	6930(3)	9296(3)	2385(3)	17.1(7)
C6	5656(4)	8852(3)	1483(3)	18.8(7)
C7	6047(4)	9532(3)	3488(3)	18.0(7)
C8	4746(4)	9111(3)	2622(3)	20.7(7)
N5	11023(3)	9900(2)	2086(2)	15.9(6)
N6	10672(3)	8588(2)	3146(2)	14.3(5)
C9	12067(3)	10059(3)	3008(3)	15.4(6)
C10	11926(3)	9106(3)	3204(3)	15.2(6)
C11	9805(3)	9364(3)	2034(3)	16.3(7)
C12	9620(3)	8387(3)	2183(3)	15.1(6)
N7	10366(3)	6628(2)	3091(2)	16.8(6)
N8	7929(3)	5016(2)	1674(2)	15.4(6)
C13	10180(3)	5905(3)	2175(3)	18.0(7)

Table S18. Fractional Atomic Coordinates (×10[4]) and Equivalent Isotropic Displacement Parameters (Å[2]×10[3]) for PipReO$_4$-II. U_{eq} is defined as 1/3 of the trace of the orthogonalised U_{IJ} tensor.

Atom	x	y	z	U(eq)
C14	8858(3)	5510(3)	1357(3)	16.0(6)
C15	8140(3)	5725(3)	2628(3)	17.6(7)
C16	9487(3)	6135(3)	3421(3)	18.1(7)
N9	7808(3)	3216(2)	2007(2)	16.8(6)
N10	7251(3)	1935(2)	3053(2)	14.9(6)
C17	8743(3)	3424(3)	3009(3)	16.7(7)
C18	8567(3)	2482(3)	3237(3)	17.5(7)
C19	6306(3)	1701(3)	2030(3)	17.7(7)
C20	6523(4)	2663(3)	1845(3)	20.9(7)
N11	11134(3)	1735(2)	1906(2)	15.1(6)
N12	13691(3)	3275(2)	3003(2)	16.7(6)
C21	11867(3)	2098(3)	1390(3)	16.0(6)
C22	13253(3)	2457(3)	2042(3)	17.7(7)
C23	13018(3)	2875(3)	3525(3)	17.2(7)
C24	11616(3)	2524(3)	2909(3)	16.8(7)

Table S19. Crystal data and structure refinement details for PhPipBF$_4$.

Identification code	PhPipBF4
Empirical formula	$C_{20}H_{29}BF_4N_4$
Formula weight	412.28
Temperature/K	120.0(2)
Crystal system	monoclinic
Space group	$P2_1/n$
a/Å	10.0061(3)
b/Å	20.8365(6)
c/Å	10.7055(3)
α/°	90
β/°	114.1070(10)
γ/°	90
Volume/Å^3	2037.34(10)
Z	4
ρ_{calc}g/cm^3	1.344
μ/mm^{-1}	0.106
F(000)	872.0
Crystal size/mm^3	0.369 × 0.172 × 0.152
Radiation	MoKα (λ = 0.71073)
2Θ range for data collection/°	4.604 to 63.146
Index ranges	-14 ≤ h ≤ 14, -30 ≤ k ≤ 30, -15 ≤ l ≤ 15

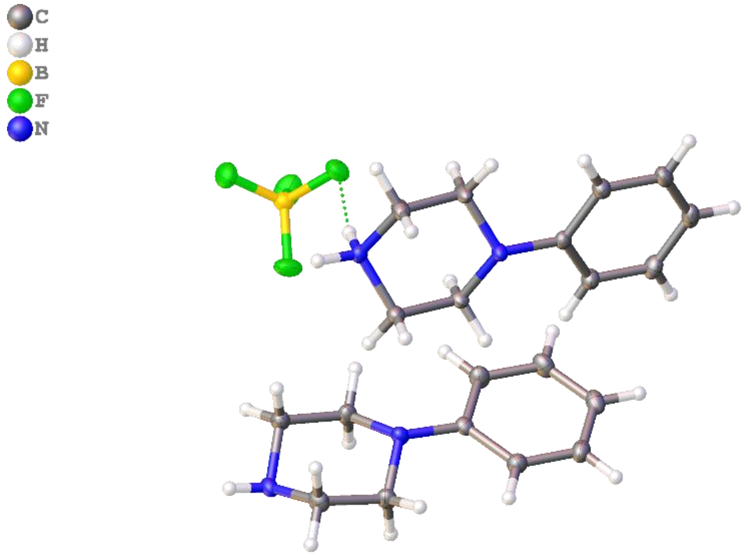

Reflections collected	57169
Independent reflections	6802 [R_{int} = 0.0536, R_{sigma} = 0.0363]
Data/restraints/parameters	6802/0/273
Goodness-of-fit on F^2	1.056
Final R indexes [I>=2σ (I)]	R_1 = 0.0443, wR_2 = 0.0903
Final R indexes [all data]	R_1 = 0.0702, wR_2 = 0.1018
Largest diff. peak/hole / e Å^{-3}	0.31/-0.22

Table S20. Fractional Atomic Coordinates (×10^4) and Equivalent Isotropic Displacement Parameters (Å^2×10^3) for PhPipBF_4. U_{eq} is defined as 1/3 of the trace of the orthogonalised U_{IJ} tensor.

Atom	x	y	z	U(eq)
F2	6825.7(9)	4195.1(4)	721.0(8)	27.85(18)
F4	6440.2(9)	3301.8(4)	-606.2(8)	29.21(18)
F1	7041.8(10)	3217.7(4)	1681.3(8)	32.15(19)
F3	8736.0(8)	3550.4(5)	904.8(9)	37.0(2)
N2	5512.6(11)	5098.4(4)	3583.8(10)	16.11(19)
N4	8184.7(10)	3057.4(5)	5855.4(10)	15.92(19)
N1	4557.9(11)	4026.4(5)	1724.9(10)	16.22(19)
N3	7386.1(11)	1760.8(5)	4924.2(10)	18.3(2)
C5	6365.4(12)	5623.0(5)	4346.1(11)	15.7(2)
C15	8976.9(12)	3580.1(5)	6659.9(12)	15.7(2)
C3	6169.0(13)	4461.4(5)	3990.3(12)	17.4(2)
C14	7513.6(13)	2623.0(6)	6514.0(13)	20.0(2)
C16	8749.9(13)	3793.8(6)	7802.4(12)	19.7(2)
C6	7616.9(13)	5531.7(6)	5560.7(12)	19.2(2)
C8	8009.1(14)	6677.8(6)	5860.7(13)	22.2(2)
C4	5124.8(13)	3926.5(5)	3227.9(12)	17.8(2)
C11	8888.6(14)	2713.4(6)	5085.5(13)	21.1(2)
C18	10460.4(13)	4670.8(6)	8177.3(13)	22.5(3)
C13	6538.0(13)	2129.1(6)	5519.5(13)	21.7(2)
C10	5958.7(14)	6257.8(6)	3909.1(13)	20.5(2)
C7	8416.2(14)	6054.7(6)	6303.5(13)	22.3(2)
C20	9999.7(13)	3918.7(6)	6323.2(13)	21.7(2)
C9	6772.6(14)	6774.4(6)	4656.8(13)	22.5(2)
C17	9478.7(14)	4331.6(6)	8537.8(13)	22.1(2)
C1	3823.4(14)	4661.7(6)	1367.1(13)	23.6(3)
C12	7898.3(15)	2205.8(6)	4144.7(13)	22.0(2)
C19	10712.1(14)	4456.0(6)	7068.6(14)	24.5(3)
C2	4882.4(15)	5192.7(6)	2101.7(12)	25.3(3)
B1	7284.6(15)	3554.8(6)	674.5(14)	18.0(2)

Table S21. Crystal data and structure refinement details for PhBorox.

Identification code	PhBorox
Empirical formula	$C_{20}H_{33}B_3F_4N_4O_5$
Formula weight	517.93
Temperature/K	120.0(2)
Crystal system	monoclinic
Space group	$P2_1/c$
a/Å	23.9300(12)
b/Å	7.7612(4)
c/Å	12.8505(7)
α/°	90
β/°	91.696(2)
γ/°	90
Volume/Å^3	2385.6(2)
Z	4
ρ_{calc}g/cm^3	1.442
μ/mm^{-1}	0.120
F(000)	1088.0
Crystal size/mm^3	0.124 × 0.09 × 0.073
Radiation	MoKα (λ = 0.71073)
2Θ range for data collection/°	5.11 to 54.996
Index ranges	-30 ≤ h ≤ 31, 0 ≤ k ≤ 10, 0 ≤ l ≤ 16
Reflections collected	5467
Independent reflections	5467 [R_{int} = ?, R_{sigma} = 0.0416]
Data/restraints/parameters	5467/0/333
Goodness-of-fit on F^2	1.136
Final R indexes [I>=2σ (I)]	R_1 = 0.0714, wR_2 = 0.1606
Final R indexes [all data]	R_1 = 0.0838, wR_2 = 0.1672
Largest diff. peak/hole / e Å^{-3}	0.36/-0.30

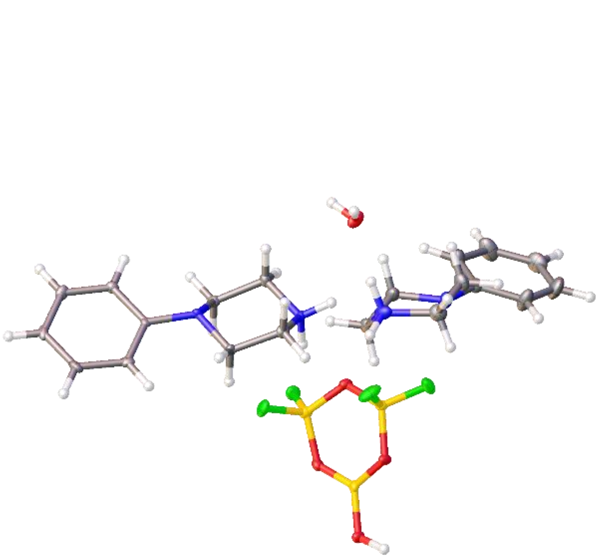

Table S22. Fractional Atomic Coordinates (×10^4) and Equivalent Isotropic Displacement Parameters (Å^2×10^3) for PhBorox. U_{eq} is defined as 1/3 of the trace of the orthogonalised U_{IJ} tensor.

Atom	x	y	z	U(eq)
F1	7596.4(7)	9474(2)	6349.8(13)	16.4(4)
F2	8419.2(7)	9749(2)	5518.7(14)	19.5(4)
F3	7037.9(8)	11314(3)	3237.8(14)	24.4(4)
F4	7977.1(8)	11508(2)	3108.4(14)	25.4(5)
O1	7595.8(9)	10759(2)	4705.8(15)	13.5(4)

Table S22. Fractional Atomic Coordinates (×10[4]) and Equivalent Isotropic Displacement Parameters (Å[2]×10[3]) for PhBorox. U_{eq} is defined as 1/3 of the trace of the orthogonalised U_{IJ} tensor.

Atom	x	y	z	U(eq)
O2	7762.4(8)	7703(2)	4933.8(15)	12.7(4)
O3	7565.8(9)	8810(3)	3218.0(15)	13.1(4)
O4	7676.9(9)	5786(3)	3530.3(16)	15.0(4)
B1	7838.8(13)	9432(4)	5358(2)	11.2(6)
B2	7543.4(13)	10589(4)	3589(2)	11.6(6)
B3	7669.2(13)	7476(4)	3880(2)	11.0(6)
O5	7054.0(9)	5801(3)	6191.0(16)	18.7(5)
N1	6729.9(10)	2521(3)	5626(2)	18.3(5)
N2	5574.6(10)	2214(4)	6096(2)	17.8(5)
C1	6279.7(13)	2798(4)	4810(3)	22.4(7)
C2	5749.0(13)	3431(4)	5296(3)	21.2(7)
C3	6536.2(13)	1435(4)	6498(2)	18.4(6)
C4	5997.7(13)	2135(4)	6932(2)	18.6(6)
C5	5007.9(13)	2345(4)	6382(2)	16.6(6)
C6	4848.6(14)	2665(5)	7395(3)	25.3(7)
C7	4286.2(15)	2660(5)	7637(3)	31.0(8)
C8	3876.3(14)	2422(5)	6885(3)	26.9(7)
C9	4031.3(15)	2126(5)	5873(3)	32.0(9)
C10	4589.7(14)	2057(5)	5628(3)	29.9(8)
N3	8400.7(9)	3612(3)	4735.8(17)	9.9(4)
N4	9488.1(9)	3584(3)	5754.5(17)	8.9(4)
C11	8496.4(12)	4384(4)	5796(2)	12.2(5)
C12	8976.7(11)	3481(4)	6359(2)	10.5(5)
C13	8923.8(11)	3636(4)	4136(2)	11.3(5)
C14	9404.4(11)	2775(4)	4734(2)	10.6(5)
C15	9990.5(11)	3187(3)	6305(2)	8.6(5)
C16	10401.9(11)	2112(3)	5892(2)	11.1(5)
C17	10909.2(11)	1829(4)	6441(2)	12.4(5)
C18	11012.7(11)	2588(4)	7398(2)	11.7(5)
C19	10604.5(12)	3650(4)	7811(2)	13.0(6)
C20	10105.0(11)	3952(4)	7279(2)	10.7(5)
